# HLA class I-naturally presented synovial tissue peptides are recognized by CD8+ T lymphocytes from rheumatoid arthritis patients

**DOI:** 10.3389/fimmu.2026.1843318

**Published:** 2026-07-03

**Authors:** Diego Catalán, Daniela Schneider, Bárbara Pesce, Miqueas Jaime, Lucero Toro, Constanza Varela-Villarroel, Darly Montano-Bruno, Lilian Soto, Francisca Bozán, María Carolina Cuéllar-Gutiérrez, Óscar Neira, Consuelo Arroyo, Guido Rivera, Milton Larrondo, Jaime Hinzpeter, Montserrat Carrascal, Juan C. Aguillón, Jaxaira Maggi

**Affiliations:** 1 Immune Regulation and Tolerance Research Group (IRT Group), Núcleo Interdisciplinario de Farmacología e Inmunología, Instituto de Ciencias Biomédicas (ICBM), Facultad de Medicina, Universidad de Chile, Santiago, Chile; 2 MED.UCHILE-FACS Lab REDECA, ICBM, Universidad de Chile, Santiago, Chile; 3 Department of Medicine, Section of Pain, Hospital Clínico de la Universidad de Chile, Santiago, Chile; 4 Division of Rheumatology, Hospital del Salvador, Universidad de Chile, Santiago, Chile; 5 Rheumatology Unit, Clínica Alemana de Santiago, Santiago, Chile; 6 Biological and Environmental Proteomics Group, IIBB-CSIC, Barcelona, Spain; 7 CSIC/Universitat Autònoma de Barcelona (UAB) Proteomics Laboratory, Universitat Autónoma de Barcelona, Cerdanyola del Vallés, Spain

**Keywords:** autoantigens, CD8+ T cells, HLA class I, immunopeptidomics, rheumatoid arthritis

## Abstract

**Introduction:**

Rheumatoid arthritis (RA) is an autoimmune disease resulting from a response driven by self-reactive CD4+ T cells that recognize autoantigenic peptides presented by antigen-presenting cells (APCs). Recent evidence suggests that CD8+ T cells are also important players in this process. This study aims to define the immunopeptidome of HLA class I molecules from RA synovial tissue (ST)- APCs and synovial fluid (SF)-pulsed monocyte-derived dendritic cells (DCs), and to prove the suitability of this approach to identify peptides recognized by CD8+ T cells from RA patients.

**Methods:**

HLA-ABC/peptide complexes were obtained from DCs generated from healthy subjects (HS), which were pulsed with a pool of RA SF (SF-DCs) or left unpulsed (UP-DCs), or obtained directly from RA ST. Isolated peptides were sequenced by mass spectrometry. The autoantigenicity of a set of ten peptides selected from this repertoire was estimated by their ability to activate CD8+ T cells from RA patients, as measured by the induction of intracellular IFN-γ expression and surface exposure of CD107a by flow cytometry.

**Results:**

Between 107 to 663 peptides were obtained from DC samples, while over 3, 500 class I peptides were identified from each ST sample. The number of peptides was narrowed down based on prioritization steps that included the selection of sequences derived from RA-relevant, immune-related proteins, for further CD8+ T-cell stimulation assays. The frequencies of CD8+ T cells co-stained for IFN-γ and CD107a were significantly higher in RA patients than in HS in response to peptides derived from the proteins MIF, ETS1, USF1, VIM, and AHR.

**Discussion:**

In the present work, we validated the use of an immunopeptidomic strategy to identify a series of novel autoantigenic CD8+ T-cell epitopes for RA, derived from synovial, mostly immune-related proteins, which may be useful for future clinical applications.

## 1 Introduction

Rheumatoid arthritis (RA) is a chronic autoimmune disease that primarily affects the joints and is characterized by synovial inflammation, with prominent infiltration of immune cells, including T and B lymphocytes, as well as antigen-presenting cells (APCs), such as dendritic cells (DCs) (1). The autoimmune process in RA is thought to be driven by DC-mediated presentation of self-antigens to CD4+ T cells, promoting the differentiation and expansion of effector and memory T helper (Th) cell subsets, including interferon (IFN)-γ-producing Th1 and interleukin (IL)-17-producing Th17 cells, which trigger the activation of infiltrating and resident joint cells, leading to progressive synovial cartilage and bone damage (2). Autoantigen recognition by autoreactive CD4+ T cells has been strongly linked to human leukocyte antigen (HLA)-DR class II risk alleles, in which the *HLA-DRB1*-encoded β-chain carries a conserved amino acid sequence known as the “shared epitope” (SE) (3, 4), located in the peptide-binding groove and influencing the presentation of self-peptides, such as citrullinated (cit) peptides (5, 6). Notably, the SE is the major genetic risk factor for RA carrying disease-specific anti-cit protein antibodies (ACPA), which are present in 70-90% of patients (5–7).

Beyond the participation of CD4+ T cells in RA, increasing evidence supports the contribution of CD8+ T cells, which recognize peptides presented by HLA class I (HLA-A, -B, and -C) molecules, and secrete cytokines and cytotoxic molecules such as perforin and granzymes (8). Results from the collagen-induced arthritis mouse model showed that the depletion of CD8+ T cells greatly reduces the incidence and/or severity of the disease (9, 10). In humans, CD8+ T cells represent around 10-35% of immune cells infiltrating the RA synovial tissue (ST) (11). Activated, cytokine-producing and cytotoxic effector or effector memory CD8+ T cells are increased in peripheral blood (PB), and especially in synovial fluid (SF), from RA patients, and their frequencies correlate with the 28-joint disease activity score (DAS-28) (12–14), while resident memory CD8+ T cells are enriched in RA synovium (15, 16). Moreover, CD8+ T cells in RA ST have been reported to produce at least as much IFN-γ as CD4+ T cells (11).

In addition, several studies showed that peripheral and synovial effector CD8+ T cells exhibit features of exhaustion, consistent with sustained antigen exposure, which are negatively correlated with DAS28 scores (13, 17–19). To further support this notion, T-cell receptor (TCR) sequencing data has suggested an antigen-driven proliferation of PB and synovial CD8+ T cells from RA patients (11, 20), and significant clonal expansion among memory CD8+ T cells has been evidenced during RA flares (21).

Furthermore, recent genetic data demonstrated a strong association between a peptide-binding groove variant of HLA-B (HLA-B*08:01; HLA-B8-Asp9) and ACPA-positive RA (22–24), as well as with anti-carbamylated protein antibodies in ACPA-negative patients (25). Besides, in a Chilean cohort, the HLA-C*07 allele was found to be enriched in RA patients versus healthy subjects (HS), while HLA-A*02:01 was among the most frequent alleles both in RA patients and HS (26). Altogether, these findings strongly point towards a role for antigen-specific CD8+ T cells in RA progression and joint destruction.

Although many RA autoantigens have been defined through autoantibody reactivity, comparatively fewer T-cell epitopes have been described, primarily related to CD4+ T cells, including peptides derived from synovial proteins, such as type-II collagen, fibrinogen, vimentin (VIM), and proteoglycan-aggrecan, mainly identified by whole-protein peptide scanning (27–30) or *in silico* HLA class II binding predictions (31). Recently, an alternative approach through immunopeptidomics, which enables the identification of HLA-bound naturally presented peptides (NPPs), accounting for antigen processing and HLA loading under physiological conditions, has allowed our group to identify six novel HLA-DR-presented CD4+ T-cell epitopes that preferentially induce proinflammatory responses in RA patients (32).

On the other hand, evidence for CD8+ T-cell targets in RA is far more limited. Pilot studies detected in RA patients a synovial expansion of CD8+ T cells specific for common virus epitopes, such as Epstein-Barr virus (EBV) antigens BMLF-1 and BZLF-1, cytomegalovirus (CMV) antigen pp65, and influenza A virus matrix protein (20, 33, 34). Autoreactive CD8+ T cells have also been reported in RA PB mononuclear cells (PBMCs), recognizing peptides from the G1 domain of aggrecan (35), as well as a broad repertoire of epitopes derived from apoptotic T-cell proteins, including VIM, heterogeneous nuclear ribonucleoprotein K, lamin B1, actin cytoplasmic 1, proteasome subunit α-1, large ribosomal protein P2, and myosin-9 (36). In addition, a recent study showed CD8+ T-cell responses, with cytotoxic and chemokine-secreting features, to cit-VIM in ACPA-positive RA patients (37).

In the present study, we applied immunopeptidomics to isolate HLA class I NPPs from RA ST-resident or infiltrating APCs, as well as from monocyte-derived DCs pulsed with RA SF. As a proof of concept, we functionally tested ten ST-derived peptides for their ability to induce CD8+ T-cell cytokine production and degranulation, five of which were preferentially recognized by PB CD8+ T cells from RA patients.

## 2 Methods

### 2.1 Patients and controls

HS, who donated buffy coats (n=4) and PB (n=25), as well as RA patients donating SF (n=15), ST (n=2), and PB (n=25), were recruited from Hospital Clínico Universidad de Chile and Hospital del Salvador (**Supplementary Tables 1**, **2**). A PB sample was also obtained from ST donors for genotyping purposes. The RA patient donor of the ST1 sample was a 58-year-old female, and parameters at the time of surgery were DAS28 4.32 (moderate disease activity), RF positive (100 UI/mL), and anti-CCP antibodies positive (>300 UI/mL). The RA patient donor of the ST2 sample was a 49-year-old male with the following parameters at the time of surgery: DAS28 4.64 (moderate disease activity), RF positive (320 UI/mL), and anti-CCP antibodies positive (898 UI/mL). The joints from which the samples were obtained were clinically swollen at the time of surgery, as determined by expert rheumatological judgment. Both patients had systemic activity indices within the active range, confirming that the samples were obtained during active synovitis rather than solely secondary degeneration. All participants signed a written informed consent according to the Declaration of Helsinki. RA patients fulfilled the American College of Rheumatology (ACR) and European League Against Rheumatism (EULAR) classification criteria for diagnosing RA (38). The Ethics Committees of each involved institution approved all procedures.

### 2.2 Blood and synovial samples

PB from HS and RA patients were processed to isolate PBMCs and to extract DNA for genotyping. Buffy coat samples from HS, carrying at least one *HLA-ABC* allele or allele group relevant for this study (HLA-A*02:01, HLA-B*08:01 or HLA-C*07) (**Table 1**), were obtained to isolate monocytes and generate DCs.

**Table 1:** HLA class I (*HLA-ABC*) alleles and HLA-ABC-bound peptide yield in synovial fluid-pulsed dendritic cell and synovial tissue samples.

Samples ^(1)^	*HLA-A* alleles	*HLA-B* alleles	*HLA-C* alleles	Number of peptides ^(2)^
UP-DC1	A*24:02 A*33:01	B*14:02 B*39:09	C*07:02 C*08:02	468
UP-DC2	A*01:01 A*26:01	B*08:01 B*38:01	C*07:01 C*12:03	513
SF-DC1	A*24:02 A*33:01	B*14:02 B*39:09	C*07:02 C*08:02	595
SF-DC2	A*01:01 A*26:01	B*08:01 B*38:01	C*07:01 C*12:03	663
SF-DC3	A*02:01 A*03:01	B*44:02 B*49:01	C*05:01 C*07:01	255
SF-DC4	A*02:01 A*34:02	B*48:01 B*58:02	C*06:02 C*08:01	107
ST1	A*01:01 A*03:01	B*35:01 B*49:01	C*04:01 C*07:01	3, 504
ST2	A*02:01 A*24:02	B*39:09 B*51:01	C*01:02 C*07:02	3, 802

(1)DC samples with the same code number derive from the same donor (2).Number of peptides obtained after discarding sequences derived from skin-specific proteins and from cell culture medium proteins. DC, Dendritic cells; UP-DC, Unpulsed DCs; SF-DC, Synovial fluid-pulsed DCs; ST, Synovial tissue.

SF samples were collected from knee joints of RA patients during therapeutic arthrocentesis. Samples were centrifuged at 480 x g for 10 min, and supernatants were incubated with hyaluronidase (100 U/mL) (Merck, Rahway, NJ, USA) at 37 °C for 60 min. After filtration, human serum albumin (HSA) and total IgG were depleted using a removal kit (Thermo Fisher Scientific). The samples were pooled together, and total protein concentration was determined by the BCA method (Thermo Fisher Scientific, Waltham, MA, USA). ST samples from RA patients were obtained from knee joints during therapeutic arthroplasty. Samples were gently minced into small fragments, and adipose tissue was removed by dissection.

### 2.3 ST processing and mononuclear cells isolation

Fresh, fractionated ST was incubated in Hanks’ balanced salt solution (HBSS), containing collagenase (200 U/mL) (Merck, Rahway, NJ, USA) at 37 °C for 18 hours, then filtered through a 70 μm nylon strainer, and washed twice with phosphate-buffered saline (PBS), by centrifugation at 400 x g for 10 min each time. Cells were resuspended in RPMI-1640 medium supplemented with 10% fetal bovine serum (FBS) (Sigma-Aldrich, St. Louis, MO, USA), and STMCs were enriched by density gradient centrifugation with Lymphoprep (Serumwerk Bernburg AG, Bernburg, Germany) for 45 min at 200 x g. The interface monolayer was collected and washed twice with PBS, followed by centrifugation at 400 x g for 10 min. Additionally, total ST samples not subjected to density-gradient enrichment were retained for phenotypic characterization. The cells were cryopreserved in FBS containing 10% dimethyl sulfoxide (DMSO) in liquid nitrogen until use.

### 2.4 Generation of DCs

DCs were generated from PB monocytes obtained from four HS and then pulsed with the RA SF pool (SF-DC1 to SF-DC4), as previously described (39). Due to the amount of material required for immunopeptidomic analysis, donor-matched unpulsed controls were available only for SF-DC1 and SF-DC2. Briefly, CD14+ monocytes were isolated from buffy coats by negative selection using the RosetteSep Human Monocyte enrichment cocktail (Stemcell Technologies, Vancouver, Canada), followed by a density gradient centrifugation using Lymphoprep. Monocytes were cultured at 2×10^6^ cells/mL in serum-free AIM-V medium (Gibco BLR, Grand Island, NY, USA) in the presence of recombinant human (rh)IL-4 and rhGM-CSF (500 U/mL each; BioLegend, San Diego, CA, USA) during 5 days at 37 °C and 5% CO_2_. On day 3, medium and cytokines were replenished, and one day later, DCs were pulsed (or not) with 200 μg/mL of the SF pool depleted of HSA and IgG. Four hours after antigen loading, cells were matured with 1 μg/mL of lipopolysaccharide (LPS; Avanti Polar Lipids Inc., Alabaster, AL, USA). Immature DCs (iDCs) were generated without LPS activation. On day 5, DCs were harvested, washed, and stored as dry pellets at -80 °C.

### 2.5 Genomic DNA extraction and HLA typing

Genomic DNA was extracted from whole blood using a salting-out procedure. DNA concentration and purity were assessed by NanoDrop spectrophotometry; yields > 100 ng/μL and A260/A280 ratios > 1.8 were considered acceptable.

Samples included in immunopeptidomic analyses underwent high-resolution HLA typing by next-generation sequencing (NGS), following the manufacturer’s instructions. Exons 2, 3, and 4 from HLA class I *loci* were amplified by multiplex polymerase chain reaction (PCR), and NGS was performed on a MiSeq platform (Illumina, San Diego, CA, USA). In contrast, PBMC donors included in functional assays were typed at the level of *HLA-A, -B*, and *-C* allele groups by low-resolution PCR using sequence-specific primers (PCR-SSP), as described by Jaime et al (26). Primer sets were selected based for HLA class I alleles/allele groups identified in samples analyzed by immunopeptidomics (**Supplementary Table 3**). PCR products were resolved on 1.5% (w/v) agarose gels with a 1Kb ladder (MaestroGen Inc.), using Tris-acetate-EDTA buffer. Bands were visualized under UV using SafeView Plus dye (Fermelo Biotec, Chile).

### 2.6 Isolation of HLA-ABC/peptide complexes

Dry DC pellets were resuspended in solubilization buffer (50 mM Tris-HCl -pH 8.0-, 150 mM NaCl, 1x Complete Protease Inhibitor Cocktail [Roche Diagnostics, Mannheim, Germany], and 1% n-dodecyl-β-D-maltoside [Merck KGaA, Darmstadt, Germany]) and sonicated (4 x 5 sec). Lysates were incubated for 2 hours at 4 °C with rotation. For ST, 500 mg of tissue samples were mechanically disrupted in solubilization buffer using zirconium oxide beads (1-mm) in a Bullet Blender tissue homogenizer (Merck, Rahway, NJ, USA). DC and ST lysates were centrifuged at 20, 000 x g for 1 hour at 4 °C. Supernatants were collected and incubated overnight (15–18 hours) at 4 °C with rotation with a pan anti-HLA-ABC monoclonal antibody (clone W6/32; Bio X Cell, Lebanon, NH, USA) coupled to CNBr-activated Sepharose beads (GE Healthcare Life Sciences, Barcelona, Spain).

After incubation, samples were loaded into Bio-Spin chromatography columns (Bio-Rad, Madrid, Spain) and washed sequentially as follows: 3x with 50 mM Tris-HCl (pH 8.0), 150 mM NaCl, 0.5% n-dodecyl-β-D-maltoside; 3x with 50 mM Tris-HCl (pH 8.0), 150 mM NaCl; 1x with 50 mM Tris-HCl (pH 8.0), 0.5 M NaCl; 3x with 50 mM Tris-HCl (pH 8.0), 150 mM NaCl; and 2x with 20 mM Tris-HCl (pH 8.0). Peptides were eluted with 0.25% trifluoroacetic acid (TFA) and stored at -80 °C.

### 2.7 Liquid chromatography-tandem mass spectrometry analysis

Eluted peptides were evaporated to dryness using a SpeedVac and reconstituted in 2.5% TFA. Samples were desalted using C18 ZipTips (Merck KGaA) and fractionated by strong cation-exchange (SCX) chromatography using GELoader epTIPS (Eppendorf, Hamburg, Germany) packed with PolySULFOETHYL A resin. Three elution steps with increasing NH_4_Cl concentrations (25, 75, and 125 mM) in 30% acetonitrile (ACN)/0.1% formic acid (FA) were applied. The fractions were desalted, evaporated to dryness, and reconstituted in 5% methanol/0.5% TFA for MS analysis. Samples were analyzed by LC-MS/MS using either an LTQ Orbitrap Velos (Thermo Fisher Scientific) or an Orbitrap Exploris 480 mass spectrometer (Thermo Fisher Scientific) coupled to an ACQUITY UPLC M-Class system (Waters, Milford, MA, USA). Data were acquired in data-dependent acquisition mode using HCD fragmentation and a Top Speed method.

### 2.8 Database search and peptide identification

LC-MS/MS spectra were searched using the SEQUEST algorithm (Proteome Discoverer v3.0, Thermo Fisher Scientific) with the following parameters: precursor mass tolerance of 20 ppm, fragment ion mass tolerance of 0.02 Da, and dynamic methionine oxidation (+15.995 Da). The search database consisted of the human UniProt protein database (release 06/2021) combined with a common contaminants database (MaxQuant). Final peptide identifications were filtered to peptide rank 1, high confidence (1% false discovery ratio at the peptide level), and search engine rank 1.

### 2.9 Bioinformatic analysis

The theoretical binding affinity of the peptides was predicted for the HLA-A, -B and -C allotypes of interest using NetMHCpan v4.2 (https://services.healthtech.dtu.dk/services/NetMHCpan-4.2/). Peptides were classified as strong binders (SB; ≤ 0.5% rank), weak binders (WB; ≤ 2% rank) or non-binders (NB; > 2% rank) for a given allotype.

To compare peptide distribution across UP-DCs and SF-DCs, peptide relative abundance values were normalized across samples and visualized descriptively using GraphPad Prism. Peptides not detected in a given sample were assigned to a value of zero for visualization. Peptides were grouped into three pattern-based categories according to their detection and relative abundance across conditions: predominantly detected in SF-DCs, predominantly detected in UP-DCs, or detected in both conditions. This grouping was used as a descriptive filtering step and not as an inferential statistical comparison.

The PANTHER Classification System (www.pantherdb.org) was used to categorize the parental proteins of HLA class I–associated peptides according to Gene Ontology (GO) biological process terms. Subcellular localization and tissue specificity of parental proteins were obtained from UniProt (https://www.uniprot.org) using the Retrieve/ID mapping tool (subcellular location and transmembrane annotations) and from The Human Protein Atlas (THPA) (https://www.proteinatlas.org) using the Tissue Atlas. Proteins reported as highly expressed in ST, SF, fibroblast-like synoviocytes or articular cartilage, or increased in serum from RA patients were considered RA tissue/cell enriched.

PubMed (Advanced Search Builder; https://pubmed.ncbi.nlm.nih.gov/advanced) was used to retrieve evidence linking parental proteins to RA, including reports describing them as targets of autoantibodies and/or T-cell responses in RA, or as enriched in RA-relevant tissues/fluids. The searches combined the terms (1) “rheumatoid arthritis” AND the “protein name”, and (2) “rheumatoid arthritis” AND “synovial” AND “proteomics”.

### 2.10 Isolation of PBMCs

Blood samples were diluted 1:1 with PBS supplemented with 2% FBS, and then a density gradient was generated with Lymphoprep, centrifuging at 400 x g for 20 min at 20 °C, using low acceleration and no brake. The PBMC-enriched monolayer was recovered, washed and centrifuged at 150 x g for 10 min to remove platelets. Cells were frozen in 10% DMSO and 90% FBS in liquid nitrogen.

### 2.11 CD8+ T-cell stimulation assay

The peptides were synthesized by NovoPep Limited (Shanghai, China) and individually tested for reactivity. Cryopreserved PBMCs were rapidly thawed, washed, and rested overnight in RPMI-1640 medium supplemented with 10% FBS prior to stimulation. PBMCs (5×10^5^ cells/well, 96-well round-bottom plates) were stimulated with 50 µg/mL individual peptides (1% DMSO final concentration) for 6 hours at 37 °C and 5% CO_2_. Anti-CD28 and anti-CD107a-PE antibodies (4 µg/mL each; BioLegend) (**Supplementary Table 4**) were added at the beginning of the culture. Brefeldin A (BFA) (BioLegend) was added at a concentration of 10 μg/mL after two hours to inhibit protein trafficking. PBMCs were left unstimulated in 1% DMSO as a negative control. As a positive control, PBMCs were stimulated with anti-CD3/anti-CD28 coated beads (Thermo Fisher Scientific); both controls were cultured in the presence of BFA. In addition, two viral peptide pools were used as stimulation controls: pool HLA-A*02 (PHLA-A*02), including peptides with high affinity for HLA-A*02:01 molecules, derived from the EBV lytic protein BMLF1 (EBV-BMLF1), influenza A virus matrix protein (IFV-Matrix), and CMV pp65 protein (CMV-pp65), and pool HLA-B*08 (PHLA-B*08), with high affinity for HLA-B*08:01 molecules, including peptides derived from the influenza virus nucleoprotein (IFV-NP) and the EBV nuclear antigen 3A (EBV-EBNA3A) (**Supplementary Table 5**). These peptides have the potential to stimulate CD8+ T cells, since most of the population has been exposed to or vaccinated against these viruses (40). After 6 hours of culture, IFN-γ expression and/or surface CD107a exposure in CD8+ T cells was evaluated by flow cytometry to measure antigen-specific cell activation and functionality (41, 42).

### 2.12 Flow cytometry analysis

#### 2.12.1 DCs staining

iDCs, SF-DCs and UP-DCs were recovered, washed in PBS, and placed at 3x10^5^ cells per well in 96-well V-bottom plates. Cells were then incubated with the Zombie UV viability dye (BioLegend), diluted 1:1000 in PBS for 15 min at room temperature. Afterwards, the cells were washed twice with PBS, centrifuging at 830 x g for 2 min each time. Surface staining was then performed using a mixture of the following anti-human monoclonal antibodies (**Supplementary Table 4**): CD11c AF647, CD86 PE, CD40 PECy7, CD14 FITC, HLA-DR AF700 and HLA-ABC APC (from BioLegend, BD Biosciences, and Thermo Fisher Scientific). Antibodies were incubated in a final volume of 100 µL of Cell Staining Buffer (BioLegend) for 30 min, at 4 °C, protected from light. Upon completion, the cells were washed twice with 100 µL of Cell Staining Buffer, centrifuging at 830 x g for 2 min each time, and finally resuspended in 200 µL of Cell Staining Buffer for acquisition. A fluorescence minus one (FMO) control was included for each fluorophore, except for HLA-ABC staining, where an isotype control antibody was used (Thermo Scientific).

#### 2.12.2 CD8+ T-cell staining

PBMCs were recovered, washed in PBS, and placed at 2x10^5^ cells per well in 96-well V-bottom plates. Cells were then incubated with the Zombie UV viability dye and washed twice with PBS. Surface staining was then performed for CD3 and CD8a using anti-human monoclonal antibodies conjugated to APC and FITC, respectively (**Supplementary Table 4**). Antibodies were incubated in a final volume of 100 µL of Cell Staining Buffer for 30 min, at 4 °C, protected from light. Then, cells were washed with Cell Staining Buffer and centrifuged at 530 x g for 2.5 min. Subsequently, the cells were resuspended in Fixation/Permeabilization buffer (Thermo Fisher) for 30 min at 4 °C, and incubated in 100 µL with a PE-Cy7 anti-human IFN-γ monoclonal antibody (**Supplementary Table 4**) for 30 min at 4 °C. Cells were washed with Cell Staining Buffer and centrifuged at 530 x g for 2.5 min. Finally, cells were resuspended in 200 µL of Cell Staining Buffer for acquisition. A FMO control was included for each fluorophore.

#### 2.12.3 ST cells and STMCs staining

Cells were thawed at 37 °C in 10 mL RPMI-1640 and washed once with Cell Staining Buffer by centrifugation at 450 x g for 5 min before staining. Then, cells were plated in 96-well V-bottom at a density of 1x10^6^/200 µl of Cell Staining Buffer. APC-conjugated anti-HLA ABC antibody or its isotype control were then added to each well, and the plates were incubated at 4 °C for 30 min in the dark. Finally, the cells were washed twice with Cell Staining Buffer and resuspended in 250 µl of the same buffer for immediate flow cytometry acquisition.

All flow cytometry assays were performed on a BD LSR Fortessa X-20 flow cytometer (Becton Dickinson BD Biosciences, Canaan, CT, USA) equipped with FACSDiva software (BD Biosciences) and analyzed using the FlowJo v10.4 software (Treestar Inc., Ashland, OR, USA).

### 2.13 Statistical analysis

GraphPad Prism 10.0.0 for Windows (GraphPad Software, Boston, MA, USA) and IBM SPSS Statistics 29.0.2.0 were used for statistical analysis and graphing. CD8+ T-cell responses were assessed by calculating the ratio between the percentage of IFN-γ+, CD107a+, or IFN-γ+CD107a+ CD8+ T cells stimulated with each peptide or positive control pools, relative to the unstimulated control. RA patients were classified as responders to a peptide when their stimulated/unstimulated IFN-γ+CD107a+CD8+ T-cell ratio exceeded a threshold equivalent to the 75th percentile (P75) of the HS group ratio distribution for that peptide. Comparisons between RA patients and HS were performed using the Mann-Whitney U test for continuous variables and Fisher’s exact test for categorical variables. For multiple comparisons, the Friedman non-parametric test with *post hoc* analysis was used. Correlations were assessed by two-tailed Pearson’s correlation tests after confirming normality with the Shapiro–Wilk test. P-values < 0.05 were considered statistically significant.

## 3 Results

### 3.1 HLA-ABC immunopeptidome from RA synovial APCs or SF-pulsed DCs

For the isolation of HLA-ABC-bound peptides, samples from RA ST, as well as HS-derived DCs pulsed or unpulsed with an RA SF pool were used. Analysis of HLA-ABC expression in both, total ST and STMCs showed high surface levels of these molecules (**Supplementary Figure 1**). Likewise, both UP-DCs and SF-DCs displayed high expression levels of HLA-ABC, HLA-DR, CD86, and CD40, unlike non-activated iDCs (**Supplementary Figure 2**), indicating that exogenous SF loading did not alter the maturation state of DCs. These results supported the feasibility of analyzing the HLA class I-associated immunopeptidome in both sample types.

**Figure 1:**
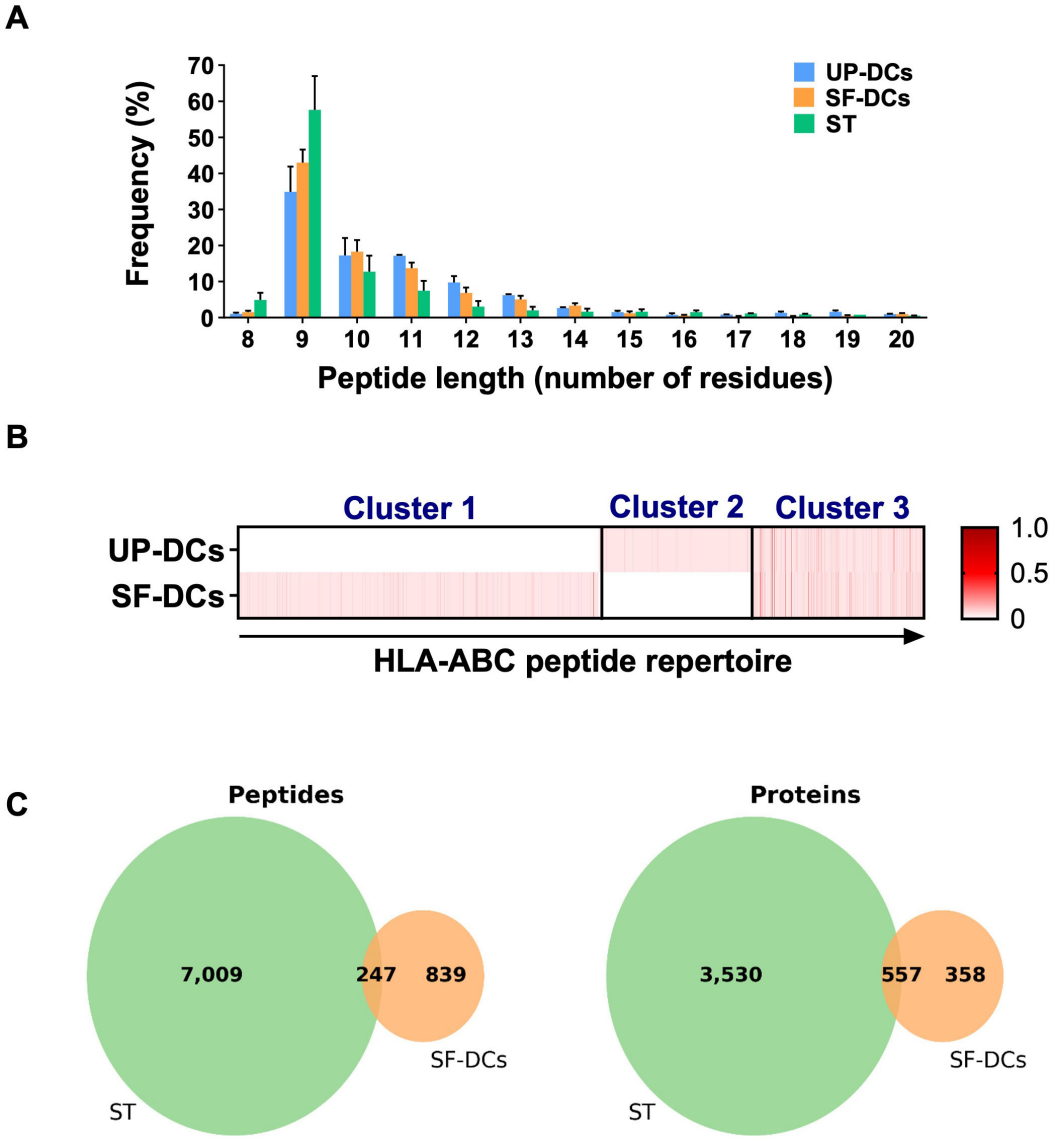
Characterization of peptides isolated from HLA class I (HLA-ABC) molecules. HLA-ABC/peptide complexes were isolated from monocyte-derived DCs generated from healthy subjects and either left unpulsed (UP-DCs) or pulsed with a pooled RA synovial fluid sample (SF-DCs), as well as from RA synovial tissue (ST) samples processed directly after tissue disruption. **(A)** Peptide length distribution in UP-DCs, SF-DCs, and ST samples. Bars represent the average peptide length frequency (%) for all donors. **(B)** Heatmap showing the relative abundance of peptides identified from UP-DCs and SF-DCs. Peptides were assigned to three descriptive clusters according to detection and relative abundance patterns across conditions: predominantly SF-DC-associated, predominantly UP-DC-associated, or detected in both conditions. White indicates non-detected peptides (relative abundance = 0) and increasing red intensity indicates higher relative abundance. This analysis was used to identify peptides preferentially associated with SF-pulsed DCs for downstream filtering. **(C)** Venn diagrams showing the overlap of filtered HLA-ABC-bound peptides and parental proteins identified in ST and SF-DCs.

The identity of peptides isolated from HLA-ABC molecules was defined based on the fragmentation spectra obtained by LC-MS/MS sequencing. Sequences derived from skin-specific proteins or from cell culture medium components were considered contaminants and excluded from further analyses. Between 107 and 663 peptides were isolated from DC samples, whereas 3, 504 and 3, 666 peptides were identified from the two ST samples analyzed (**Table 1**). To avoid overinterpreting differences in peptide yield between biologically distinct sample types, ST and DC-derived samples were not considered as quantitatively comparable inputs. ST samples were directly processed tissue specimens, whereas SF-DCs were monocyte-derived DCs pulsed with a pooled RA SF sample. Thus, ST was used to capture an *in situ* synovial HLA class I peptide repertoire, while SF-DCs were used as a complementary model to explore peptides generated after DC processing of soluble synovial material. The number of peptides identified from DC samples increased with the initial number of DCs analyzed. This trend was statistically evaluated for all DC samples and for SF-DCs alone, reaching significance in the SF-DC subset (**Supplementary Figure 3**). A separate UP-DC-only correlation was not performed because only two UP-DC samples were available. In all samples, the most frequent peptide length corresponded to 9 amino acids, consistent with the expected size of canonical HLA-ABC ligands (**Figure 1A**). A comparison of NPPs obtained from UP-DCs and SF-DCs showed marked differences in the peptide repertoire (**Figure 1B**). Although these differences could be explained by the SF load, they may be also influenced by a limitation to detect less abundant sequences.

To focus on peptides more likely to derive from synovial proteins, only sequences detected exclusively in SF-DCs (cluster 1 in **Figure 1B**), together with those identified in ST samples, were considered for subsequent analyses. After discarding sequences derived from HLA-ABC molecule chains and those shorter than nine and larger than twelve residues, according to the HLA-class I classically defined peptide core, a final count of 1, 086 and 7, 256 peptides, deriving from 915 and 4, 087 parental proteins, was reached for SF-DC and ST samples, respectively (**Table 2**). A comparison of these filtered datasets showed that 247 peptides and 557 parental proteins were shared between SF-DC and ST samples, whereas 839 peptides and 358 proteins were exclusive to SF-DCs, and 7, 009 peptides and 3, 530 proteins were exclusive to ST samples (**Figure 1C**).

**Table 2:** Number of 9-12mer HLA-ABC-bound peptides and parental proteins.

	SF-DC1	SF-DC2	SF-DC3	SF-DC4	ST1	ST2
Peptides, *n*	342	397	253	101	3, 504	3, 802
Parental proteins, *n*	281	341	211	88	2, 441	2, 652
Total peptides ( 1) , *n*	1, 086				7, 256	
Total parental proteins^(1)^, *n*	915				4, 087	

(1)The total number indicated is not equal to the sum of peptides/proteins obtained per sample, as some of them were found in more than one sample. SF-DC, Synovial fluid-pulsed dendritic cells; ST, Synovial tissue.

**Figure 2:**
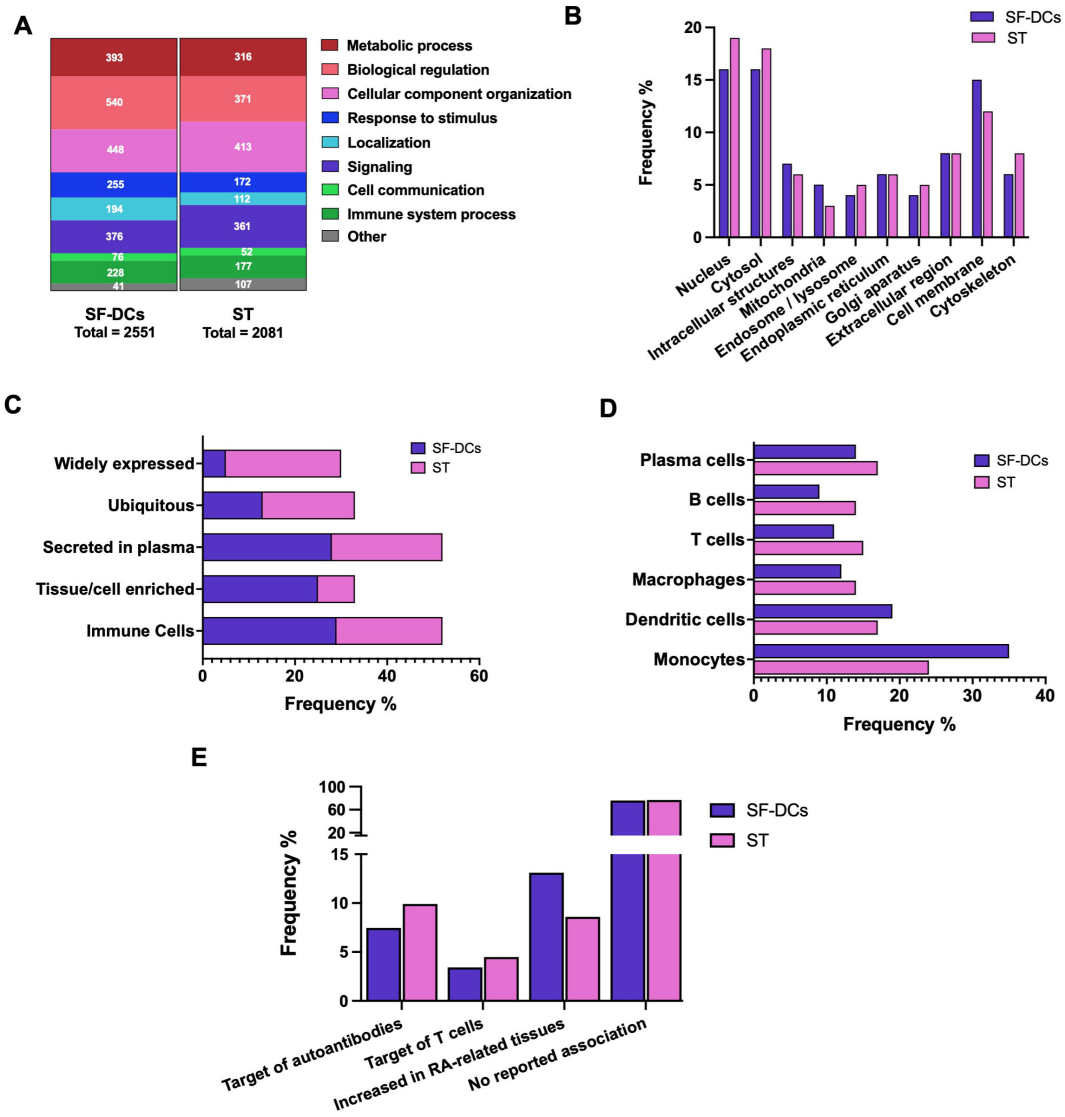
Characterization of parental proteins from which HLA-ABC-associated peptides were identified. **(A–C)** Frequency distribution of 874 and 983 parental proteins, identified immunopeptidome analysis of synovial fluid-pulsed dendritic cells (SF-DCs) and synovial tissue (ST) samples, respectively, according to: **(A)** Gene Ontology biological process annotations, **(B)** Uniprot and THPA subcellular localization annotations, and **(C)** THPA expression pattern classification. **(D, E)** Frequency distribution of immune cell-associated parental protein according to: **(D)** THPA expression pattern across major immune cell subsets, and **(E)** Literature curation and database annotation reports of association with RA. Proteins could be assigned to more than one category.

Afterwards, to characterize the origin and function of the parental proteins identified in the HLA-ABC peptide repertoire, UniProt and THPA annotation tools were applied to 874 proteins detected in SF-DCs but not detected in UP-DCs, and to 983 proteins reproducibly detected in both ST samples. GO analysis revealed that these proteins were associated with a wide range of biological processes, with the most frequently represented categories corresponding to metabolic processes, biological regulation, cellular component organization, signaling, and immune system processes (**Figure 2A**). In addition, subcellular localization analysis showed that parental proteins were predominantly annotated to intracellular compartments, including nucleus, cytosol, and cytoskeleton, which is expected for the HLA class I-processing pathway, as well as to the cell membrane and extracellular region (**Figure 2B**). According to the THPA expression pattern classification, a substantial proportion of proteins were either secreted into plasma or preferentially expressed in immune cells, whereas smaller fractions corresponded to ubiquitously expressed proteins or to proteins enriched in specific tissues or cell types (**Figure 2C**). Given the prominence of immune cell-associated proteins, a more detailed analysis was conducted, revealing that these proteins were most frequently linked to monocytes, with a broadly comparable representation across DCs, plasma cells, B and T cells, and macrophages (**Figure 2D**). Finally, to investigate the reported description of the parental proteins expressed in immune cells (**Figure 2C**) as RA-associated proteins, a literature curation (PubMed) and database annotation examination was performed. From this search, it could be estimated that, for SF-DCs and ST samples, approximately 7% and 10% of proteins had been previously described as target of autoantibodies, 3% and 5% as targets of T-cell responses, and 13% and 9% as being overexpressed in tissues related to the disease, respectively (**Figure 2E**). Notably, more than 76% of the identified parental proteins in both datasets had not been previously reported in the context of RA (**Figure 2E**), highlighting a large number of potentially novel immune cell-associated antigenic targets unearthed by this immunopeptidomic approach.

### 3.2 Selection of HLA-ABC NPPs for T-cell stimulation assays

The next aim was to stimulate CD8+ T cells from RA patients with a limited number of peptides as a proof of principle that the previously described strategy is suitable for the identification of autoantigenic CD8+ T-cell epitopes for this disease. For this purpose, a stepwise prioritization strategy was applied to our repertoire to establish a set of ten peptides, derived from RA-related proteins, with a plausible probability to elicit self-reactive CD8+ T-cell responses (**Supplementary Figure 4**).

First, the set was restricted to 9-mers, since peptides of this length are the most abundantly presented by MHC class I molecules and are classically selected for immunogenicity prediction studies (43, 44). In parallel, sequence-based physicochemical rules were applied to favor motifs compatible with stable HLA class I binding and to avoid highly charged or structurally unfavorable candidates. These rules included constraints around P1/P2/PΩ (P9) and charge distribution. This peptide-level filtering reduced the dataset to 2, 046 sequences.

Next, the binding affinity was predicted for each peptide against the complete set of HLA-A, -B, and -C molecules expressed by the corresponding sample donors (monocyte donors for SF-DCs, or ST donors). Peptides were prioritized when predicted to bind at least three HLA-ABC allotypes present in their sample of origin, increasing the likelihood of them being presented to CD8+ T cells. In addition, prioritized peptides included those with high predicted affinity to at least one disease-relevant or study-relevant allele or allele group: HLA-B*08:01, given its association with RA (22–24), *HLA-C07*, due to its association with RA in a Chilean cohort (26), and HLA-A*02:01, since this allele is among the most frequent HLA class I alleles in several populations, including RA patients from the Chilean cohort (26). This HLA-binding prioritization reduced the dataset to 1, 514 sequences.

Afterwards, peptides derived from proteins with evidence of relevance to RA (i.e., reported autoantibody reactivity, T-cell reactivity, and/or increased levels in RA-related tissues/fluids) were selected. This yielded over one hundred parental proteins of interest. To maximize disease specificity, the selection was narrowed down to 34 peptides by prioritizing 26 non-housekeeping parental proteins that are inflammation-regulated or have functional relevance in RA (**Supplementary Table 6**).

Finally, the selection was further refined using a higher-stringency mechanistic criteria, prioritizing proteins supported by strong evidence for a causal or amplifying role in RA pathogenesis (e.g., key nodes in inflammatory circuits such as Th17 regulation, synovial inflammation, or pathways whose inhibition reduces inflammation in RA). In addition, to cover different levels of immunological evidence, the panel included one reported RA autoantibody target with limited characterization (ANP32A) (45), and a well-established and extensively validated autoantigen in RA (VIM) (30, 46). This process yielded a shortlist of 15 peptides derived from 10 different proteins (**Supplementary Table 6**). When more than one peptide from the same protein met these criteria, a single peptide was selected for functional assays, resulting in a final shortlist of 10 peptides (**Table 3**). To facilitate interpretation of this final panel, the corresponding parental proteins were annotated according to the categories used in **Figure 2**, including biological process, subcellular localization, expression pattern, and RA-related functional relevance (**Supplementary Table 7**). This set represents a mechanistically prioritized panel for functional validation, rather than the ten most abundant or statistically ranked proteins in the dataset. All these peptides were identified in ST, and one of them, derived from XPO1, was also detected in SF-DCs. For simplicity, peptides are hereafter referred to by the corresponding parental protein gene name or the commonly used protein abbreviation reported in Uniprot.

**Table 3:** HLA class I-presented peptides selected for immunostimulatory assays.

Parental protein	Peptide name	Sequence	Donor HLA class I molecule(s) to which the peptide is predicted to bind	Study-relevant HLA class I molecule(s) to which the peptide is predicted to bind
Acidic leucine-rich nuclear phosphoprotein 32 family member A	ANP32A	FLSTINVGL	A*02:01 (SB); B*39:09 (WB); C*01:02 (WB)	A*02:01 (SB)
Aryl hydrocarbon receptor	AHR	ILPPQLALF	A*02:01 (WB); A*24:02 (SB); C*01:02 (SB); C*07:02 (WB)	A*02:01 (WB); C*07 (SB)
Cathepsin D	CATD	YLSQDTVSV	A*02:01 (SB); B*39:09 (WB); C*01:02 (WB); C*07:02 (WB)	A*02:01 (SB)
Exportin-1	XPO1	VLIDYQRNV	A*02:01 (SB); C*01:02 (WB) A*02:01 (SB); C*05:01 (WB); C*07:01 (WB)	A*02:01 (SB)
Macrophage migration inhibitory factor	MIF	FLSELTQQL	A*02:01 (SB); A*24:02 (WB); B*39:09 (SB); C*01:02 (SB); C*07:02 (SB)	A*02:01 (SB); B*08:01 (SB); C*07 (SB)
Progressive ankylosis protein homolog	ANKH	SISDVIAQV	A*02:01 (SB); B*51:01 (WB); C*01:02 (WB)	A*02:01 (SB); C*07 (SB)
Protein C-ets-1	ETS1	ILWEHLEIL	A*02:01 (SB); B*39:09 (WB); C*01:02 (WB); C*07:02 (WB)	A*02:01 (SB); B*08:01 (WB)
Raftlin-2	RFTN2	SILDIVTKV	A*02:01 (SB); B*51:01 (WB); C*01:02 (WB); C*07:02 (WB)	A*02:01 (SB); C*07 (SB)
Upstream stimulatory factor 1	USF1	FPDPNVKYV	A*01:01 (WB); B*35:01 (SB); C*04:01 (SB); C*07:01 (WB)	A*02:01 (WB); B*08:01 (WB); C*07 (SB)
Vimentin	VIM	SLQEEIAFL	A*02:01 (SB); C*01:02 (SB); C*07:02 (WB)	A*02:01 (SB)

The peptide name corresponds to the parental protein gene symbol or commonly used protein abbreviation according to UniProt. Each peptide was assigned as strong binder (SB) or weak binder (WB) to HLA class I molecules carried by the sample donor and for the study-relevant HLA class I molecules HLA-A*02:01, HLA-B*08:01, and HLA-C*07 according to the NetMHCpan v4.2 classification.

### 3.3 HLA-ABC NPPs derived from ST samples elicit functional CD8+ T-cell responses in RA patients

CD8+ T-cell activation in response to the set of 10 peptides was evaluated by flow cytometry using IFN-γ production and CD107a (LAMP-1) surface exposure as functional readouts (**Supplementary Figures 5**–**7**). CD8+ T cell-derived IFN-γ is key in inflammatory damage in RA (8), whereas CD107a surface expression reflects degranulation upon stimulation (42), and both are commonly used as indicators of antigen-driven CD8+ T-cell activation.

**Figure 3:**
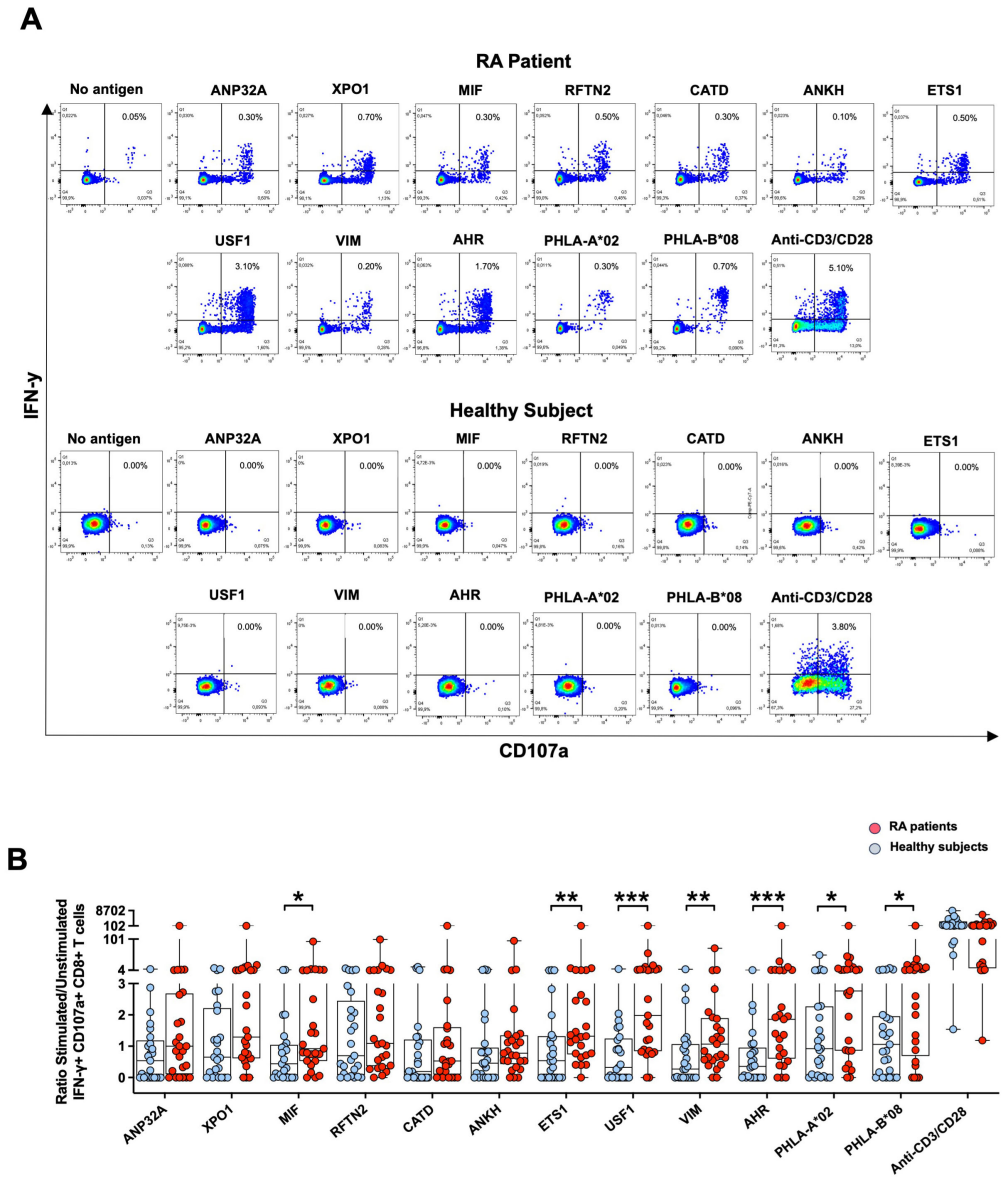
Combined CD8+ T-cell IFN-γ and cytotoxic responses to HLA class I-presented peptides. **(A)** Representative flow cytometry plots from one rheumatoid arthritis (RA) patient and one healthy subject (HS) showing the co-staining of IFN-γ and CD107a in gated live, singlet CD3+CD8+ T cells under unstimulated and peptide-stimulated conditions, including viral peptide pools (PHLA-A*02 and PHLA-B*08) and anti-CD3/anti-CD28 beads positive controls. Percentages indicate the frequency of IFN-γ+CD107a+ cells within CD8+ T cells. **(B)** Graph showing the ratio of IFN-γ+CD107a+CD8+ T cells in stimulated over matched unstimulated cultures for each peptide in RA patients and HS (Mann-Whitney U test). The Friedman non-parametric test was performed to compare peptide-specific IFN-γ+CD107a+CD8+ T-cell response ratios within the RA group, with *post hoc* analysis. Boxes and whiskers indicate the median and range, respectively. *p < 0.05, **p < 0.01, ***p < 0.001.

Immunostimulatory assays resulted in significantly higher peptide-stimulated/unstimulated ratios of IFN-γ+CD8+ T cells in RA patients compared to HS for the USF1 (p < 0.01) and ANP32A peptides (p < 0.05; **Supplementary Figure 6B**). On the other hand, CD107a+CD8+ T-cell ratios were significantly higher in RA patients than in HS for the XPO1, ETS1, AHR (p < 0.05), and USF1 peptides (p < 0.01; **Supplementary Figure 7B**). When focusing on the more stringent functional readout of IFN-γ+CD107a+CD8+ T cells (**Figure 3A**), significantly higher responses in RA patients than in HS were observed for the MIF (p < 0.05), ETS1, VIM (p < 0.01), USF1, and AHR peptides (p < 0.001; **Figure 3B**). Notably, both viral peptide pools also elicited stronger responses in RA patients than in HS (p < 0.05; **Figure 3B**), suggesting that an increased overall CD8+ T-cell effector responsiveness may contribute, at least in part, to the differences observed between groups.

To further assess whether peptide-specific T-cell responses were enriched in RA patients at the individual level, patients were classified as responders when their stimulated/unstimulated IFN-γ+CD107a+CD8+ T-cell ratio exceeded the 75th percentile of the HS group IFN-γ+CD107a+CD8+ T-cell ratio distribution for that peptide, and responder frequencies were compared between groups using Fisher´s exact tests. Under this categorical framework, CD8+ T-cell responses to USF1 (60% in RA patients vs 24% in HS, p = 0.0209) and AHR peptides (64% in RA patients vs 24% in HS, p = 0.0096) were found to be significantly higher in RA patients (**Table 4**).

**Table 4:** Percentage of rheumatoid arthritis (RA) patients and healthy subjects (HS) classified as responders to each peptide.

Peptide name	% Responder RA patients (n)	% Responder HS (n)	*P*-value
ANP32A	32 (8)	12 (3)	0.1706
XPO1	44 (11)	24 (6)	0.2321
MIF	48 (12)	24 (6)	0.1398
RFTN2	36 (9)	24 (6)	0.5380
CATD	28 (7)	24 (6)	0.9999
ANKH	44 (11)	24 (6)	0.2321
ETS1	48 (12)	24 (6)	0.1398
USF1	60 (15)	24 (6)	0.0209*
VIM	52 (13)	24 (6)	0.0792
AHR	64 (16)	24 (6)	0.0096**

RA patients were classified as responders when their stimulated/unstimulated IFN-γ+CD107a+CD8+ T-cell ratio exceeded the 75th percentile of the HS group IFN-γ+CD107a+CD8+ T-cell ratio distribution for that peptide. Fisher’s exact test. *p < 0.05, **p < 0.01.

Next, comparisons of peptide-specific IFN-γ+CD107a+CD8+ T-cell responses within the RA patient group were performed. This within-group comparison is useful to rank the relative functional prominence of peptide-specific CD8+ T-cell responses in RA but, by itself, do not address disease specificity. The USF1 peptide induced significantly higher responses than the CATD (p = 0.0027) and the ANKH (p = 0.0097) peptides (**Figure 2B**). This within-group comparison is useful to rank the relative functional prominence of peptide-specific CD8+ T-cell responses in RA but, by itself, do not address disease specificity.

Finally, to explore whether peptide-specific CD8+ T-cell responsiveness in RA was influenced by the predicted peptide binding to HLA-ABC molecules, RA patients with at least four known *HLA-ABC* allele groups (out of six possible) were classified, for each peptide, according to whether they carried at least one HLA-ABC molecule predicted to bind that peptide as strong binder (SB+ versus SB-). Responder and non-responder frequencies were then compared between SB+ and SB- RA patients for each peptide (**Table 5**; **Supplementary Table 8**). No consistent differences were found in the responsiveness to peptides between both groups of RA patients (**Table 5**). Given the stronger CD8+ T-cell responses observed for some peptides in RA patients compared to HS, the distribution of HLA-ABC molecules to which peptides are classified as SB across both groups was evaluated. A higher percentage of individuals carrying at least one HLA-ABC molecules with high affinity was observed only for the ETS1 (p = 0.0054) and ANKH peptides (p = 0.0374) in RA patients compared to HS (**Supplementary Table 9**). Altogether, these findings suggest that distinctive CD8+ T-cell responses are not solely explained by the predicted binding capacity of peptides to HLA-ABC molecules and may also involve differences in the CD8+ T-cell repertoire presented by RA patients and HS.

**Table 5:** Percentage of rheumatoid arthritis (RA) patients responding or not to each selected peptide, according to the presence of an HLA-ABC molecule to which a peptide is assigned as strong binder (SB).

Peptide name	% Responder RA patients		% Non-responder RA patients		*P*-value
	SB+ (n)	SB- (n)	SB+ (n)	SB- (n)	
ANP32A	22.2 (4)	0 (0)	55.6 (10)	22.2 (4)	0.5242
XPO1	38.9 (7)	0 (0)	38.9 (7)	22.2 (4)	0.1193
MIF	44.4 (8)	0 (0)	55.6 (10)	0 (0)	>0.9999
RFTN2	22.2 (4)	5.6 (1)	61.0 (11)	11.1 (2)	>0.9999
CATD	16.7 (3)	5.6 (1)	77.8 (14)	0 (0)	>0.9999
ANKH	33.2 (6)	5.6 (1)	55.6 (10)	5.6 (1)	>0.9999
ETS1	38.9 (7)	0 (0)	50.0 (9)	11.1 (2)	0.4967
USF1	61.1 (11)	0 (0)	38.9 (7)	0 (0)	>0.9999
VIM	44.4 (8)	0 (0)	50.0 (9)	5.6 (1)	>0.9999
AHR	44.4 (8)	11.1 (2)	38.9 (7)	5.6 (1)	>0.9999

RA patients were classified as responders when their stimulated/unstimulated IFN-γ+CD107a+CD8+ T-cell ratio exceeded the 75th percentile of the healthy subjects group IFN-γ+CD107a+CD8+ T-cell ratio distribution for that peptide. Each peptide was assigned as SB+ or SB- to HLA class I molecules carried by each patient according to the NetMHCpan v4.2 classification. Fisher’s exact test. n = 18.

## 4 Discussion

In recent years, the application of deep immunophenotyping methods such as single-cell RNA sequencing, mass cytometry and multispectral imaging has confirmed the association of different blood and synovial effector CD8+ T cell populations with the emergence, development, and course of RA (11, 15, 21, 47). Additionally, TCR repertoire sequencing has revealed an increased oligoclonality in CD8+ T cells from RA patients, indicative of antigen-driven clonal expansion (20, 21, 37). Nevertheless, the antigens targeted by CD8+ T cells in RA cells remain elusive.

Immunopeptidomics has been widely used for the discovery of T-cell epitopes in cancer, infections and autoimmune diseases, among other conditions (48–50). Our group and others have previously reported the feasibility of using ST, SF-DCs or PBMCs to extract HLA class II-presented peptides, among which relevant RA-specific T-cell epitopes have been identified through the detection of strong autoreactive PB CD4+ T-cell responses (32, 51–53). In this work, we have, for the first time, utilized an immunopeptidomic approach to describe a novel set of synovial, immune-associated peptides able to induce the degranulation and IFN-γ production by PB CD8+ T cells from RA patients.

We were able to isolate numerous unique HLA class I peptides from RA ST and DCs loaded with RA SF. While the ST offers a unique source of peptides being presented *in situ* by joint APCs and potentially recognized by infiltrating T cells, SF-DCs provides an alternative, scalable platform for the obtention of synovial NPPs that considers the nuances of antigen processing by these priming, cross-presenting professional APCs. As expected, both repertoires exhibited features of *bona fide* HLA class I-presented peptides, such as a predominant length of 9–12 residues, intermediate-to-high theoretical affinity for parental HLA class I allotypes, and a prevailing subcellular localization of their parental proteins compatible with the HLA class-I processing pathway. It was not surprising to find that many peptides were derived from proteins expressed by immune cells, mainly from monocytes, DCs and macrophages, since, on the one hand, monocyte-derived DCs were one of our sources of peptides and, on the other hand, HLA class I-expressing macrophages and DCs infiltrating the RA ST have been extensively described, including DC1 cells specialized in cross-presenting antigens to CD8+ T cells (54–56). Precisely, this property may account for the abundance of plasma protein-derived peptides in our list of HLA class I-presented peptides.

Although this MHC class I peptide repertoire represents a snapshot of potential antigens driving inflammation in joints, the true initiators of the autoimmune response in RA may be absent. It has been proposed that in these patients, tolerance to self-proteins is broken in the mucosa of lungs, intestine or gingiva (57), to later develop into a systemic disease. An immunopeptidomic analysis of those tissues in early RA patients, followed by T-cell activation assays, would be an interesting route to pinpoint the initial targets of CD8+ T cells. Fortunately, the improvement in MS technologies has already allowed the identification of HLA-bound peptides from scarce samples, such as bronchioalveolar lavage cells and extracellular vesicles from human fluids, facilitating this kind of studies (58, 59). The same strategy could be applied to tissues affected by extraarticular manifestations of the disease, such as rheumatoid nodules or the spleen in patients with Felty syndrome, as has been attempted in the past (60).

Since the current study was designed as a proof of principle approach with a limited cohort of patients and controls envisaged for functional assays, we restricted the number of tested peptides to an accessible number of ten. Among the vast diversity of peptides, we focused on those derived from parental proteins with well-defined RA-relevant and/or immune-related functions. While the best described self-antigens for RA correspond to structural proteins such as type-II collagen, aggrecan, or VIM (27, 29, 37), or to housekeeping enzymes, such as α-enolase or peptidyl-arginine deiminase-4 (61, 62), only a limited number of immune cell-associated proteins have been reported as targets of either antibodies or T cells in RA patients.

From our initial set of peptides tested in T-cell assays, three peptides derived from transcription factors with immune functions stand out by strongly inducing CD8+ T-cell IFN-γ and cytotoxic responses in RA patients: USF1, AHR and ETS1. Along with its role in regulating lipid metabolism and cancer progression (63, 64), USF1 is crucial for the induction of class II major histocompatibility complex expression by IFN-γ (65), and has been involved in preventing inflammaging-related defects in macrophages and in osteoclast differentiation (66, 67). Regarding AHR, this environment-sensing transcriptional regulator modulates processes such as cell differentiation, trafficking, antigen presentation and tolerance induction (68). AHR is highly expressed in RA ST (69, 70), and plays a crucial role in murine models of arthritis by driving Th17 cell differentiation, but it is also important in sustaining regulatory B-cell and Treg induction (71–73). Finally, ETS1 is a vital transcription factor for lymphoid and endothelial cell development and has been widely described as an oncogene (74). Single-nucleotide polymorphisms in the *ETS1* locus have been associated with RA (75), and critically, ETS1 is overexpressed in RA ST, with an ETS1-coordinated pathway shown to be pivotal in programming destructive synovial fibroblasts in RA (76, 77). To our knowledge, no antibody or T-cell responses have been described for USF1 or AHR in autoimmune conditions; however, a recent study identified antibodies for cit-ETS1 peptides in serum from RA patients (78).

Another peptide that induced strong IFN-γ and degranulation responses in CD8+ T cells from RA patients in this study is derived from MIF, a pleiotropic inflammatory cytokine secreted by activated leukocytes and synoviocytes, which promotes the chemotaxis of macrophages and lymphocytes (79, 80). Genetic variations of MIF have been associated with high serum MIF levels and RA severity (81). RA SF and ST are enriched in MIF (82), and studies in animal models and human synoviocytes have suggested that MIF is an essential contributor for joint destruction (83, 84). According to our search in public resources (Pubmed, AAg Atlas (85) and IEDB (86)), MIF has not yet been described as an autoantigen. Of note, HLA class I immunopeptidomic studies in healthy tissues and tumor cells have frequently sequenced the USF1 (87–90), AHR (89, 91, 92), ETS1 (88–90, 92), and MIF peptides (87–92) peptides identified here; however, no T-cell activation assays have been performed to our knowledge. In revealing an original set of T-cell epitopes involved in immune processes, our results open a new avenue that may offer a conceptual shift that departs from “classical” structural and housekeeping proteins as preferential targets of RA autoimmune responses.

A different situation applies to VIM, an intermediate filament protein, extensively described as being recognized by autoantibodies and CD4+ T cells from RA patients, especially in its cit version (30, 46, 93–95). Furthermore, two studies demonstrated the existence of RA-specific CD8+ T-cell cytotoxic and IFN-γ responses against whole cit-VIM and a VIM peptide, respectively (36, 37). Strikingly, the VIM epitope used in the work by Citro et al. (VIM_226-234_), derived from a fragment of VIM cleaved by caspases during CD8+ T-cell apoptosis and design based on its high affinity for the HLA-A2 molecule (36, 96), is identical to the sequence that triggered strong RA CD8+ T-cell activation in the present study, which was isolated from an HLA-A*02:01+ sample, thus supporting the validity of our approach for self-epitope discovery. Moreover, among our peptide repertoire obtained from an HLA-A*02:01+ ST sample, we identified several peptides derived from VIM and other apoptotic epitopes identical to those described by Citro et al. (data not shown), suggesting that APCs in the synovium may capture proteins from apoptotic cells and cross-present their immunodominant epitopes through HLA class I molecules, possibly activating local self-reactive CD8+ T cells.

Unsurprisingly, this VIM peptide has been frequently found in HLA-A*02:01+ samples by immunopeptidomic studies (89, 92, 97). Remarkably, this epitope was also found to be strongly recognized by CD8+ T cells from HLA-A2+ patients with multiple sclerosis, chronic hepatitis C, human immunodeficiency virus infection, and cardiac transplantation (96, 98–100). A follow-up study demonstrated that HLA-A2+ RA patients with high blood frequencies of CD8+ T cells specific for VIM_226–234_ and other apoptotic epitopes are more likely to respond to anti-tumor necrosis factor (TNF) therapy. Interestingly, these apoptotic epitope-specific CD8+ T cells appeared to be confined to the naïve subpopulation in HS and anti-TNF responders, but to the effector memory subpopulation in non-responder patients; these cells were also present in SF and were able to kill Tregs in a NKG2D-dependent fashion (101). In this regard, a thorough phenotypic and functional characterization of the peptide-specific CD8+ T cells identified in this work should be the subject of future studies.

It is worth noting that among the five peptides described above, four of them (excepting USF1) are theoretically SB for the HLA-A*02:01 allotype, a common HLA class I variant in the Chilean population and also among RA patients, while MIF, USF1 and AHR peptides exhibit high theoretical affinity for HLA-C*07 molecules, which are also prevalent among Chilean RA patients (26). In turn, the MIF and USF1 peptides were estimated to strongly bind to the HLA-B*08:01 molecule, a locus that has recently been linked to RA (24). Although experimental binding assays are required to confirm these observations, these results can be helpful to further explore whether the biological connection underlying the association between these HLA class I alleles and RA stems from a more favorable presentation of self-epitopes to disease-driving CD8+ T cells.

We are aware that, in applying a literature-oriented prioritization of proteins, we could disregard a multitude of peptides obtained from our immunopeptidomic strategy, which could emerge as novel self-antigens lacking a previous link with RA. To take full advantage of the possibilities offered by this dataset for the discovery of new antigens, unbiased methods can be implemented in the future. For instance, several algorithms have been developed to integrate parameters involving MHC class I presentation and TCR recognition to predict immunogenic epitopes from tumor or pathogen-derived naturally presented peptide ligands (102–106). On the other hand, the information of MHC class I eluted peptides can be combined with TCR sequences of expanded CD8+ T-cell clones in RA to model or test potential MHC-peptide-TCR interactions, as has been assayed in different models (107–109). Lastly, high-throughput T-cell epitope mapping methods can be performed, such as the use of peptide pools in matrix-based designs, measuring cytokines by ELISpot or ELISA as readout (110–112).

The systematic implementation of this approach may also pave the way for personalized antigen-specific therapeutic interventions. For instance, the identification of HLA class II self-epitopes in RA has supported the launch of clinical trials using tolerogenic DCs loaded with autoantigenic peptides, aiming at modulating pathogenic CD4+ T cells in SE+ RA patients (113–115). Similarly, promising results in pre-clinical studies of type 1 diabetes have been achieved by administering HLA class I self-epitopes either directly (116) or attempting to steer them to cross-presenting DCs (117, 118).

In summary, in the present work we have validated an immunopeptidomic strategy to identify a series of novel autoantigenic CD8+ T-cell epitopes for RA, derived from synovial, immune-related proteins, which may be useful in future clinical applications.

## 5 Conclusions

Our study presents a series of original synovial, mostly immune-related, self-epitopes able to induce strong CD8+ T-cell IFN-γ and degranulation responses in RA patients, identified using an immunopeptidomic strategy with no precedent in this field. These results can be useful for a better understanding of the role of self-reactive CD8+ T cells in RA and the influence that RA-associated HLA class I alleles exert in presenting autoantigenic peptides, and in the design of tolerogenic therapies aimed at modulating pathogenic CD8+ T-cell responses in this disease.

## Data Availability

The original contributions presented in the study are publicly available. This data can be found here: ProteomeXchange Consortium via the PRIDE partner repository, accession number PXD079646.
