# Supplementary material for: HLA class I-naturally presented synovial tissue peptides are recognized by CD8+ T lymphocytes from rheumatoid arthritis patients
Source: Front Immunol. 2026 Jul 3;17:1843318. doi: 10.3389/fimmu.2026.1843318 (PMC13375624; doi:10.3389/fimmu.2026.1843318)
Supplement: Supplementary file 1 [file SupplementaryFile1.docx]

**Supplementary Figures**

**A**


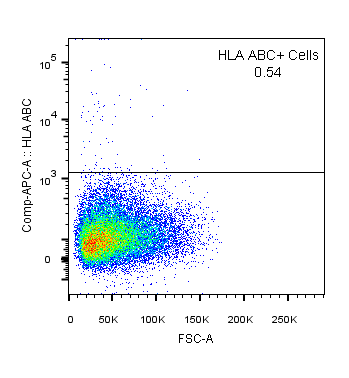

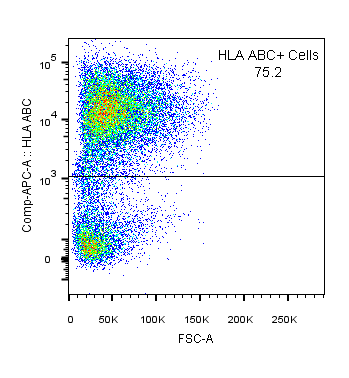

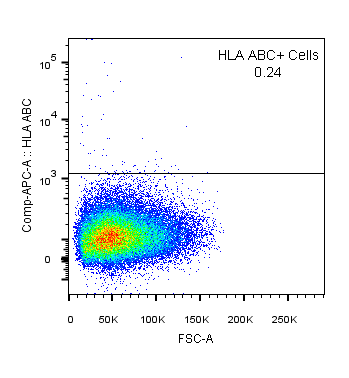


FSC-A

**ST cells**

**STMCs**

**Isotype control**

**HLA-ABC**


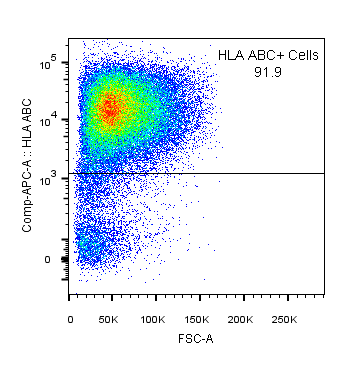


APC


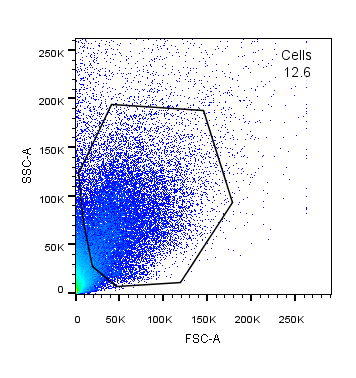

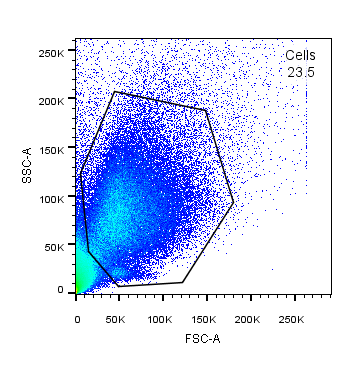


SSC-A

FSC-A

FSC-A


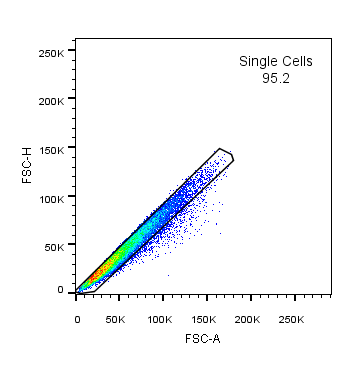

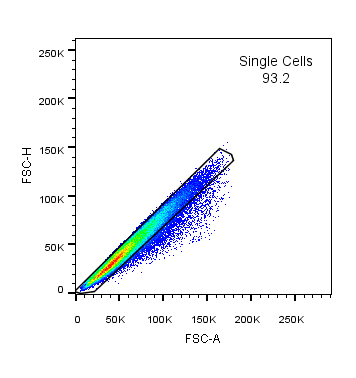


FSC-H

FSC-H

FSC-A

**HLA-ABC+ cells**

**75.2**

**Isotype+ cells**

**0.54**

**HLA-ABC+ cells**

**91.9**

**Isotype+ cells**

**0.24**

**Single cells**

**95.2**

**Single cells**

**93.2**

**Cells**

**12.6**

**Cells**

**23.5**

**B**


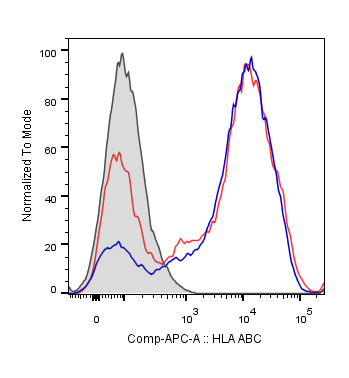


**Isotype control**

**ST cells**

**STMCs**

Normalized to mode

Mean Fluorescence Intensity (MFI)

**Supplementary Figure 1. HLA-ABC expression in total synovial tissue (ST) cells and ST mononuclear cells (STMCs) from rheumatoid arthritis patients**. Cell surface HLA-ABC expression was assessed by flow cytometry. Representative plots from one ST donor are shown (A) The gating strategy used for total ST cells (top) and STMCs (bottom) excluded debris and small, low-granularity cells generated by collagenase digestion, as well as doublets, according to size (FSC-A and FSC-H) and complexity (SSC-A) parameters. The frequency of HLA-ABC-expressing cells and an isotype control staining are shown as density plots. (B) Histogram showing HLA-ABC expression, measured as mean fluorescence intensity (MFI), in total ST cells and STMC compared to isotype control staining.


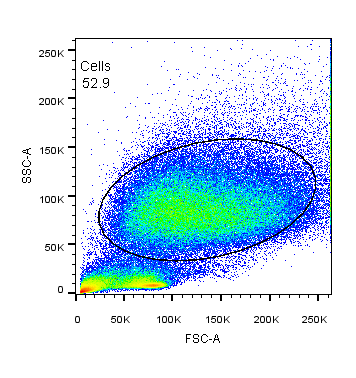

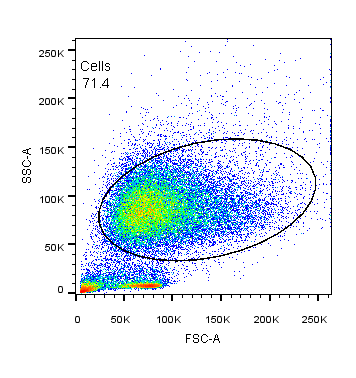

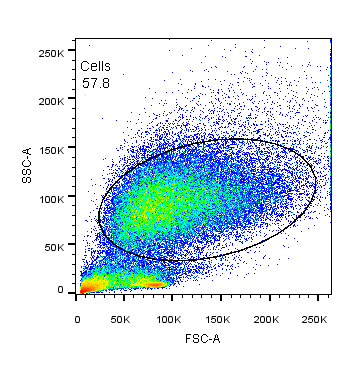

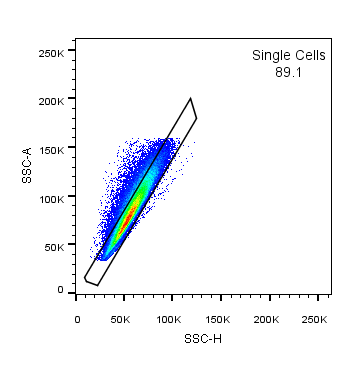

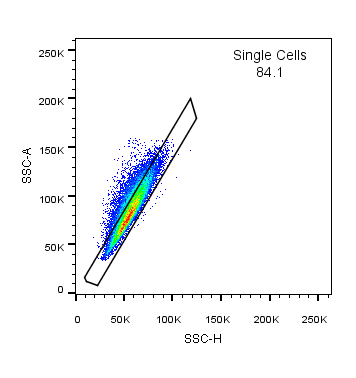

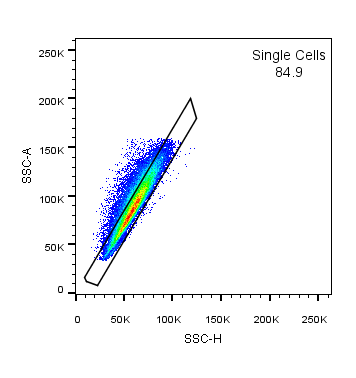

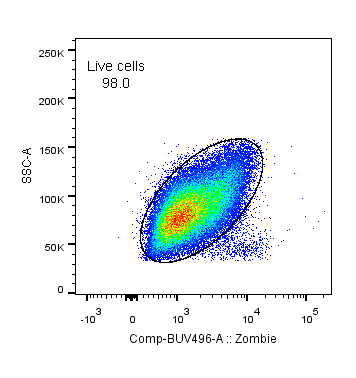

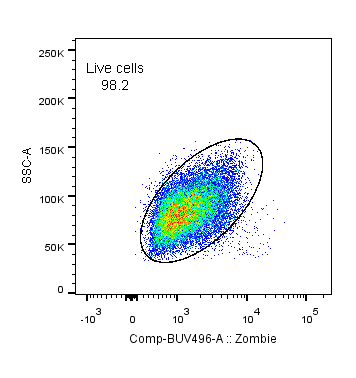

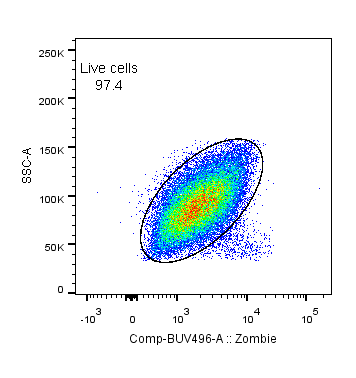

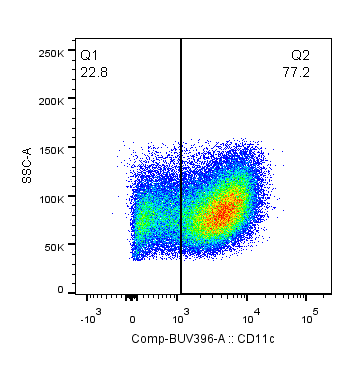

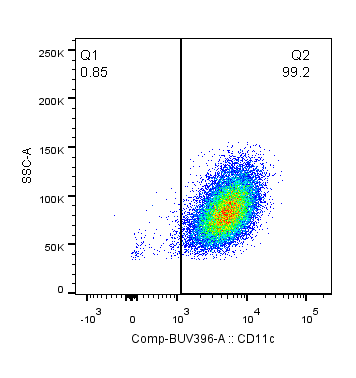

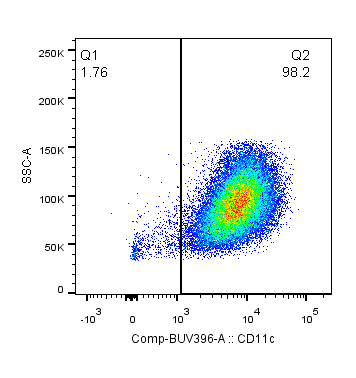


SSC-A

FSC-A

SSC-H

Viability dye

CD11c

**iDCs**

**UP-DCs**

**SF-DCs**

**A**

**B**


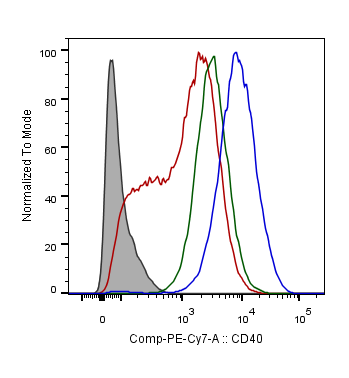

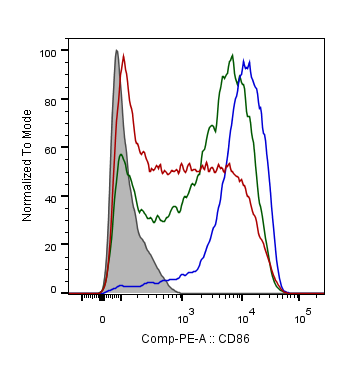

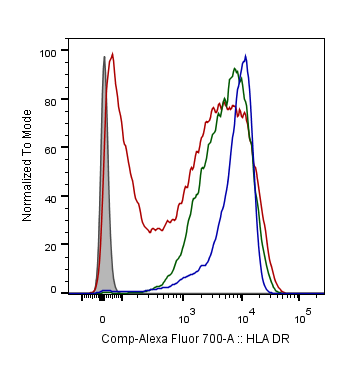

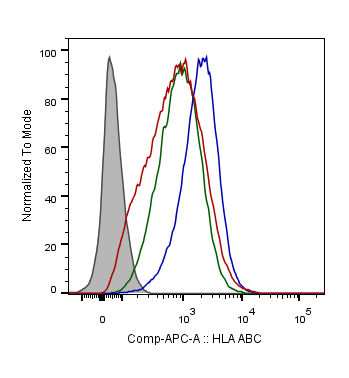


MFI

Normalized to mode

**HLA-ABC**

**HLA-DR**

**CD86**

**CD40**

**FMO/Isotype control**

**iDCs**

**UP-DCs**

**SF-DCs**

**Supplementary Figure 2. Mature dendritic cells (DCs) generated from monocytes of healthy subjects (HS) and pulsed with synovial fluid (SF) from patients with rheumatoid arthritis (SF-DCs) preserve the main phenotypic features of unpulsed DCs (UP-DCs).** Cell surface markers were assessed by flow cytometry. Representative plots from one monocyte donor are shown (A) Gating strategy for immature DCs (iDCs), mature UP-DCs and mature SF-DCs, showing the sequential identification of single cells, live cells, and CD11c+ cells. (B) Histograms showing the mean fluorescence intensity (MFI) for surface HLA-ABC, HLA-DR, CD86 and CD40 expression in iDCs, UP-DCs, and SF-DCs compared to isotype control staining (HLA-ABC), or fluorescence minus one (FMO) control (HLA-DR, CD86, and CD40).

**A**

**B**

**Peptide number**

**Peptide number**

**Supplementary Figure 3**. **Correlations between the number of peptides isolated from HLA class I (HLA-ABC) molecules and the number of DCs from where they were isolated**. HLA-ABC/peptide complexes from synovial fluid (SF)-pulsed dendritic cell (SF-DCs) samples were immunoprecipitated, and bound peptides were sequenced by liquid chromatography coupled to mass spectrometry. Unpulsed DCs (UP-DCs) were used as controls. Correlation analyses between the number of identified peptides and the initial number of DCs are shown for (A) SF-DCs and UP-DCs, and (B) SF-DCs alone. Correlations were evaluated using the Pearson’s correlation test after evaluation of normality by the Shapiro-Wilk test. A separate correlation analysis for UP-DCs alone was not performed because only two UP-DC samples were available, making a formal correlation statistically uninformative. **p < 0.01.


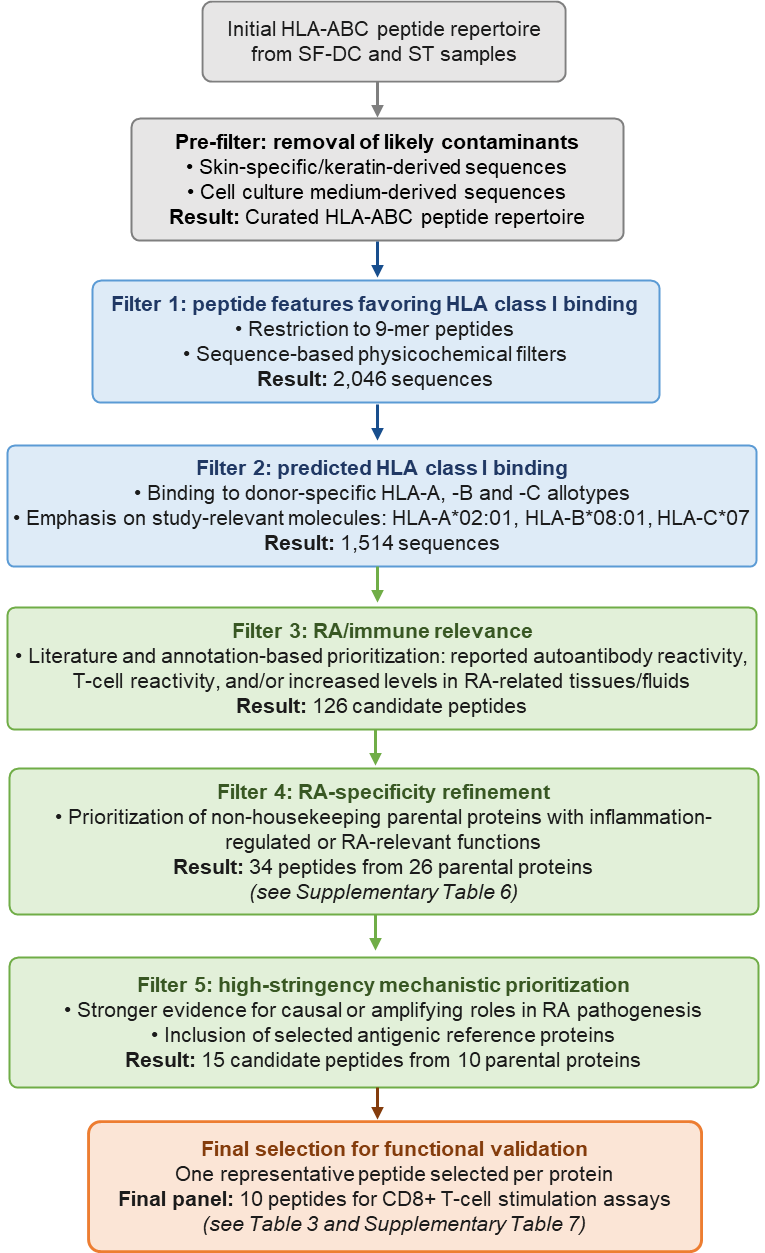


**Supplementary Figure 4. Peptide prioritization workflow for the selection of candidates used in CD8+ T-cell stimulation assays.** Schematic summary of the sequential filtering strategy applied to the HLA-ABC peptide repertoire obtained from SF-DC and ST samples. After removal of likely contaminants, including skin-specific/keratin-derived and cell culture medium-derived sequences, the curated repertoire was filtered at the peptide level according to 9-mer length, physicochemical features favoring HLA class I binding, and predicted binding to donor-specific and study-relevant HLA class I molecules. Candidate peptides were then prioritized at the parental-protein level according to RA/immune relevance, non-housekeeping status, inflammation regulation, and functional relevance in RA. A final high-stringency mechanistic prioritization step yielded 15 candidate peptides from 10 parental proteins, from which one representative peptide per protein was selected, resulting in the final panel of 10 peptides used in CD8+ T-cell stimulation assays. ST, synovial tissue; SF-DC, synovial fluid-pulsed dendritic cells; RA, rheumatoid arthritis.


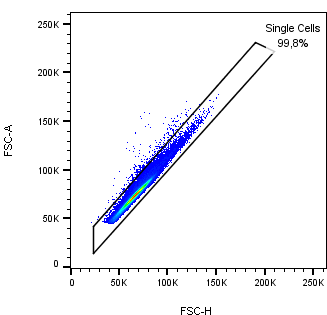

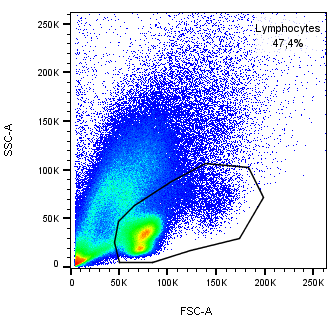

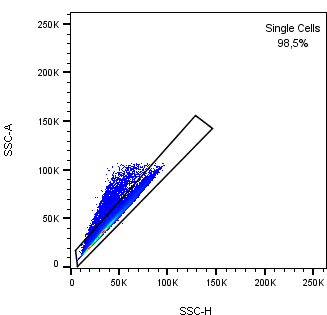


FSC-A

SSC-H

SSC-A

SSC-A

FSC-A

FSC-H


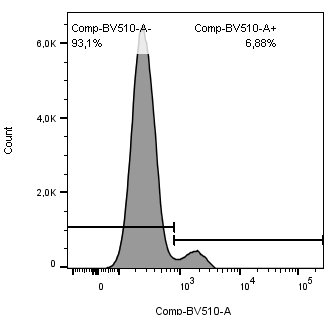


Viability dye

Count


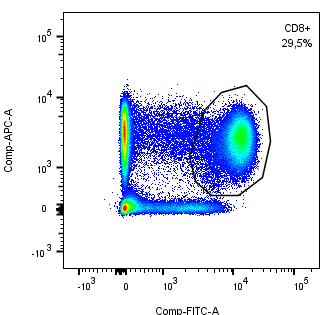


CD8

CD3


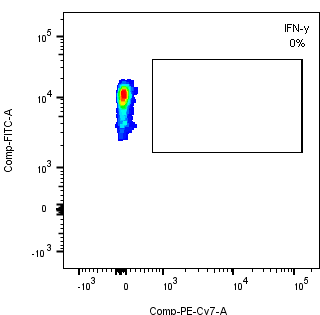


IFN-γ

CD8


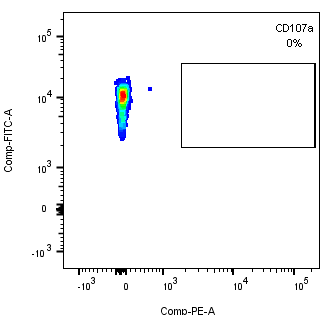


CD107a

CD8

**FMO IFN-γ**

**FMO CD107a**

**Single cells**

**99.5%**

**Single cells**

**99.8%**

**Live cells**

**93.1%**

**CD8+**

**29.5%**

**Lymphocytes**

**47.4%**

**Supplementary Figure 5.** **Gating strategy for the analysis of CD8+ T-cell activation in PBMC stimulation assays**. The lymphocyte population was selected from a dot plot according to cell size (FSC) and complexity (SSC). Subsequently, SSC-H vs SSC-A and FSC-H vs FSC-A plots were used to select single cells, allowing exclusion of cellular doublets or triplets. Dead cells were excluded using a viability dye. The CD3+CD8+ population was then selected as CD8+ T cells. Fluorescence minus one (FMO) controls were included for IFN-γ and CD107a to set the corresponding gates.

**CD8**

**IFN-y**


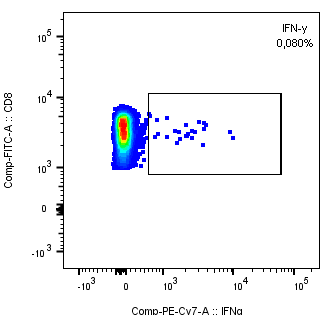

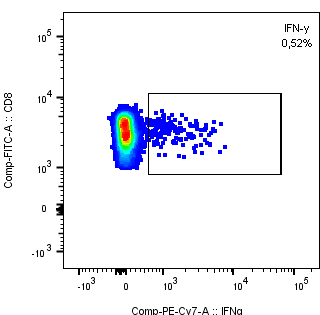

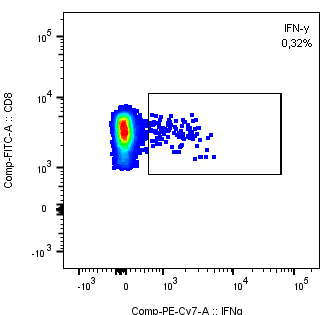

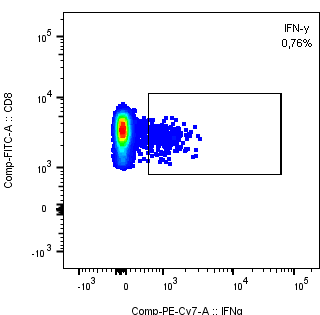

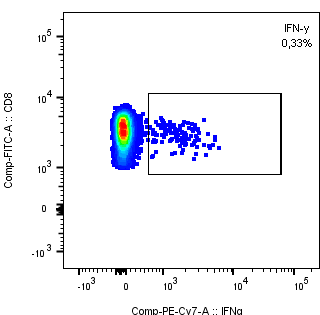

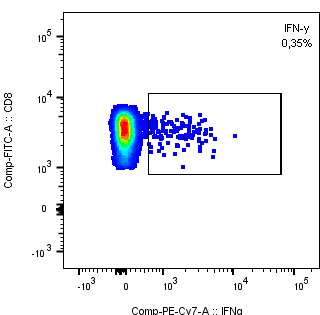


**CATD**

**No antigen**

**ANP32A**

**XPO1**

**MIF**

**RFTN2**

**RA Patient**

**ANKH**

**ETS1**


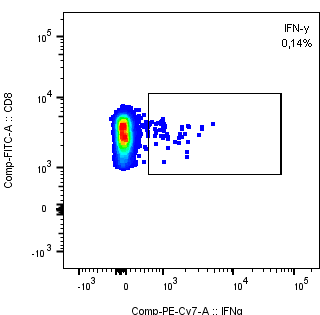


**Anti-CD3/CD28**

**PHLA-A*02**

**PHLA-B*08**

**USF1**

**VIM**

**AHR**


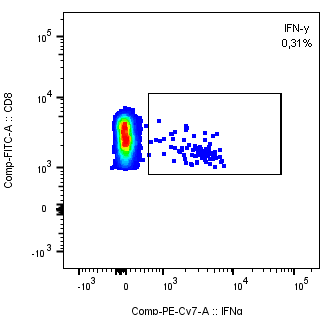

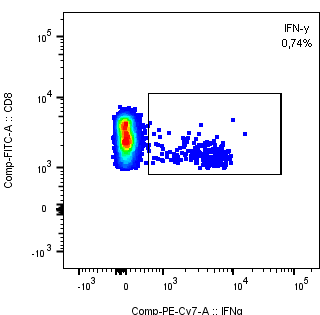

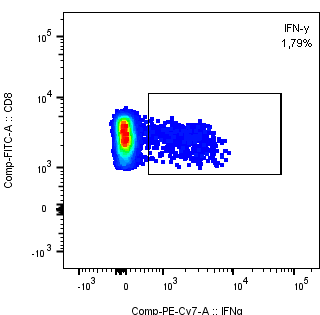

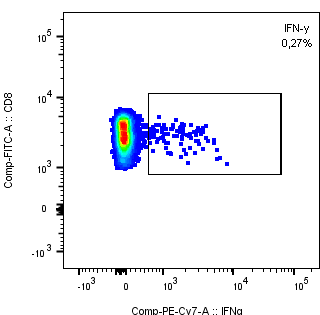

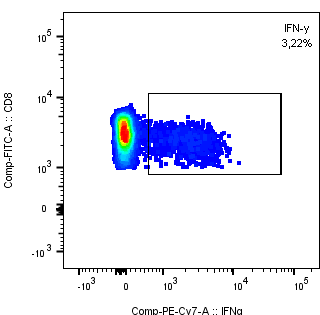

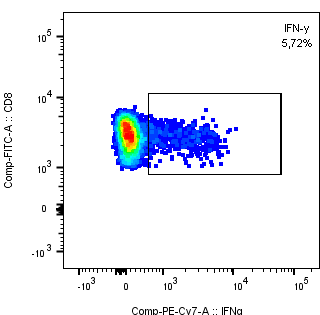

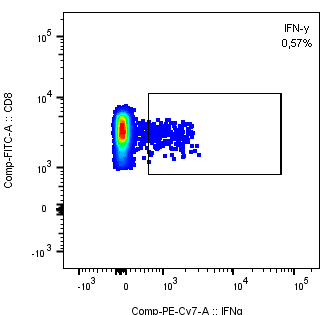


**CATD**

**PHLA-A*02**

**PHLA-B*08**

**No antigen**

**ANP32A**

**XPO1**

**MIF**

**RFTN2**

**USF1**

**VIM**

**AHR**

**Healthy Subject**


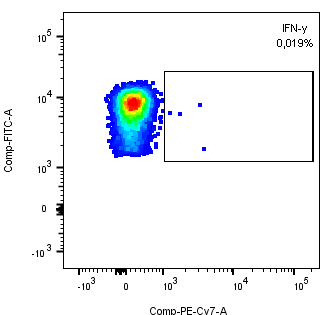

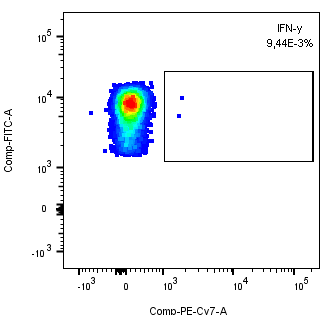

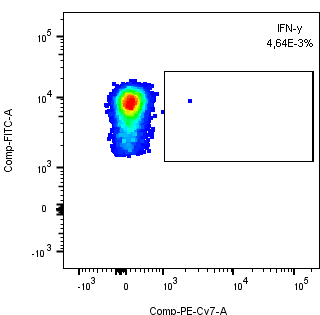

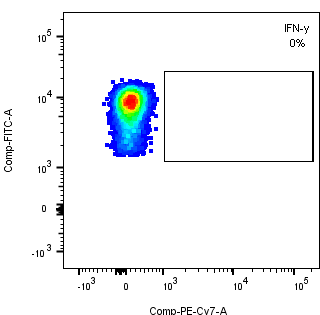

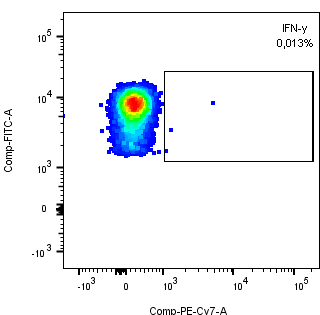

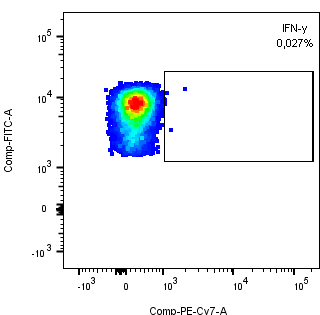

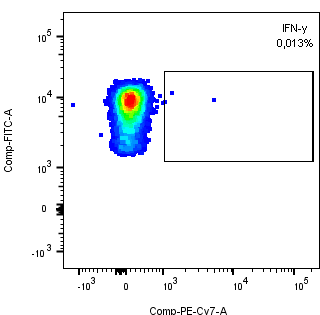

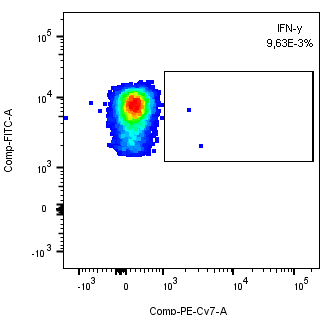

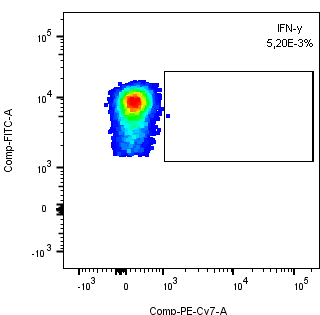

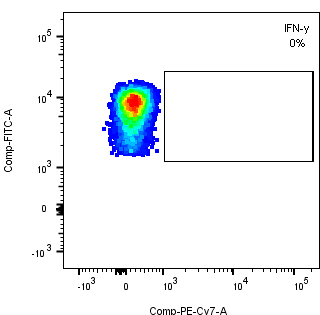

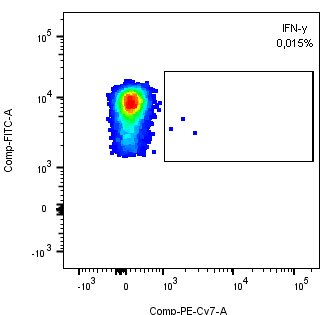


**ANKH**

**ETS1**


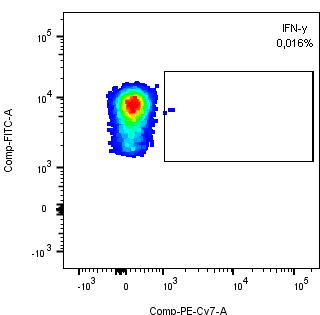

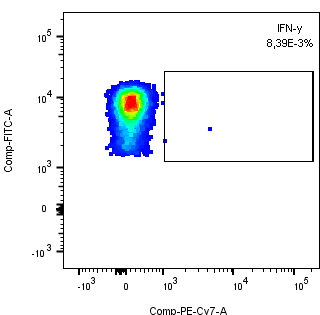


**Anti-CD3/CD28**


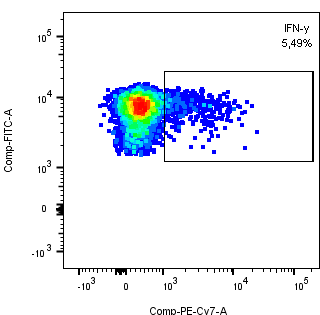


**A**


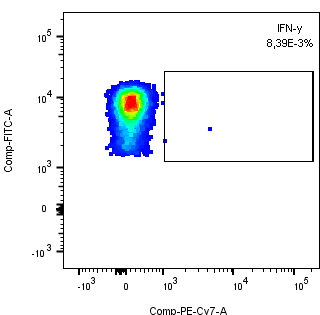


**ETS1**

**B**

**RA patients**

**Healthy subjects**

**Supplementary Figure 6. CD8+ T-cell IFN-γ responses to HLA class I-presented peptides**. (A) Representative flow cytometry plots showing IFN-γ expression in gated live, singlet CD3+CD8+ T cells from one rheumatoid arthritis (RA) patient and one healthy subject (HS) under unstimulated and peptide-stimulated conditions. (B) Graph showing the ratio of CD8+ T cells expressing IFN-γ in stimulated over matched unstimulated cultures for each peptide. Boxes and whiskers indicate the median and range, respectively. Anti-CD3/anti-CD28 coated beads were used as a positive control. Two viral peptide pools were used as stimulation controls: PHLA-A*02 and PHLA-B*08, with high affinity for HLA-A*02:01 and HLA-B*08:01 molecules, respectively. Group comparisons were performed using Mann-Whitney U test. *p < 0.05; **p < 0.01.

**A**

**CD8**

**CD107a**

**CATD**

**No antigen**

**ANP32A**

**XPO1**

**MIF**

**RFTN2**

**RA Patient**

**ANKH**

**ETS1**

**Anti-CD3/CD28**

**PHLA-A*02**

**PHLA-B*08**

**USF1**

**VIM**

**AHR**


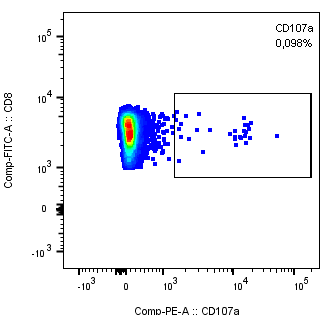

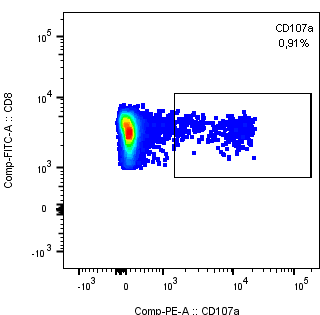

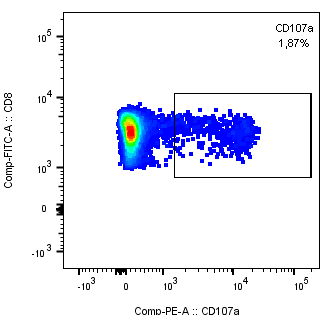

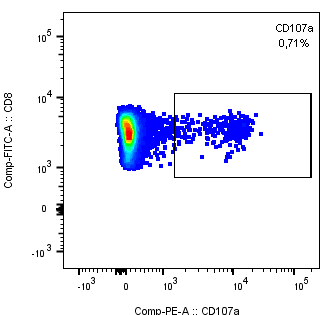

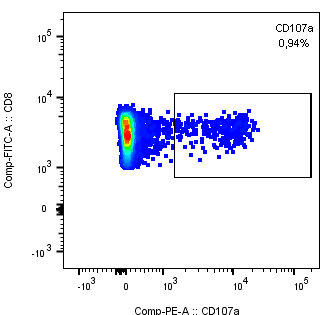

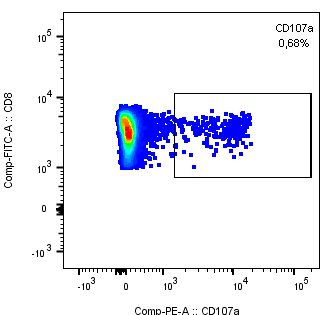

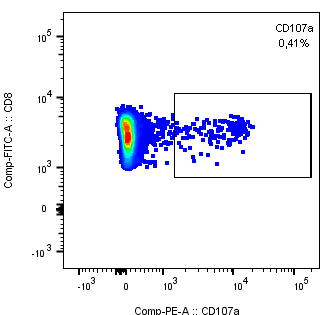

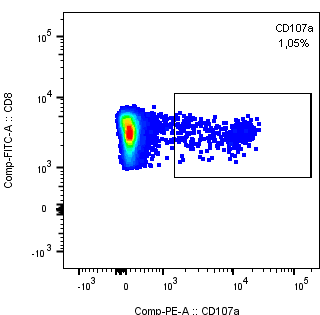

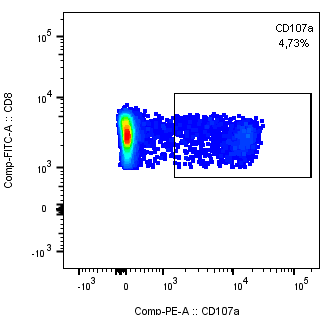

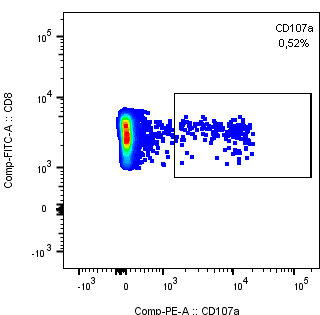

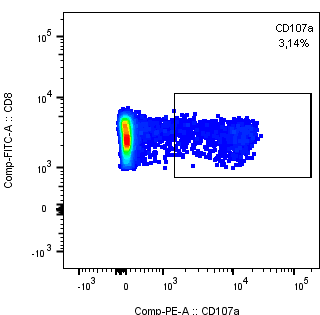

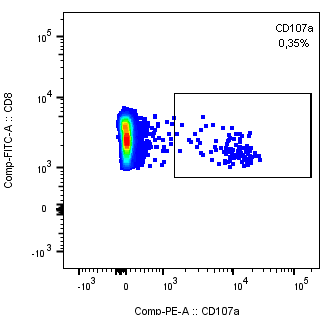

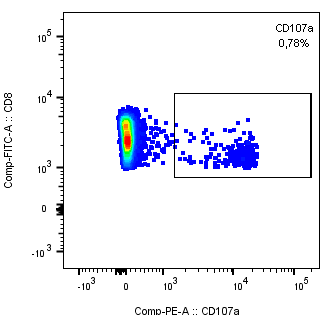

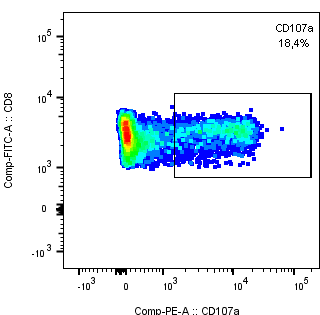


**CATD**

**PHLA-A*02**

**PHLA-B*08**

**No antigen**

**ANP32A**

**XPO1**

**MIF**

**RFTN2**

**USF1**

**VIM**

**AHR**

**Healthy Subject**

**ANKH**

**ETS1**

**Anti-CD3/CD28**


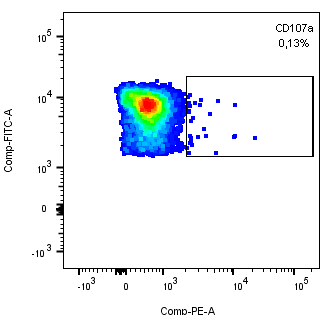

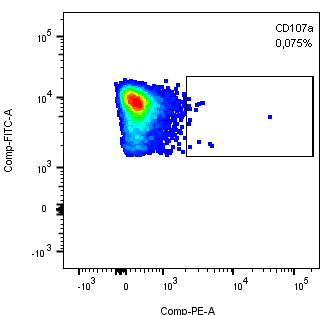

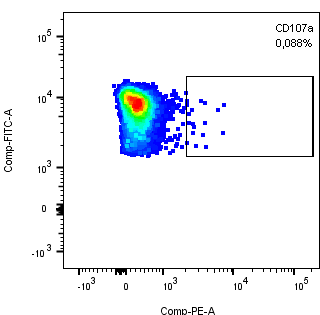

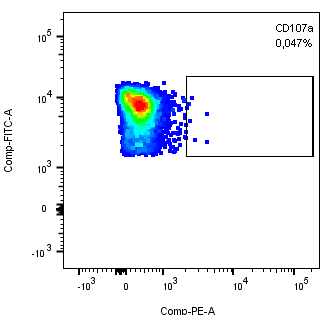

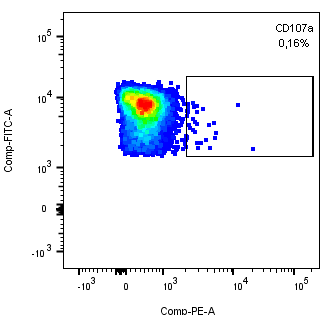

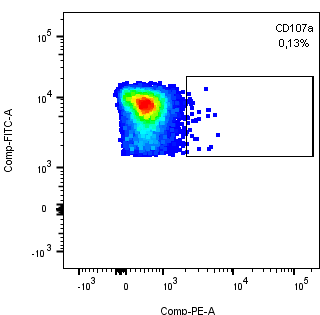

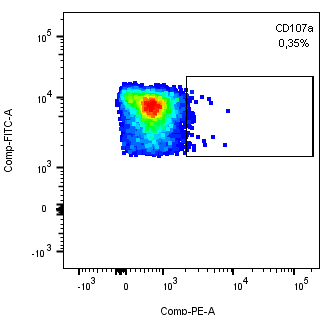

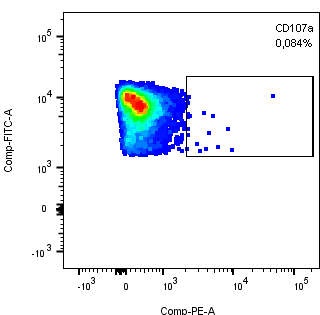

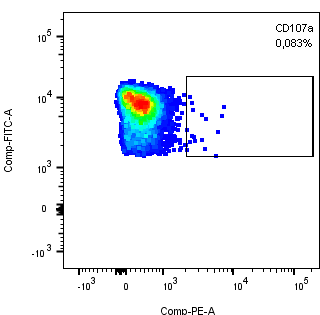

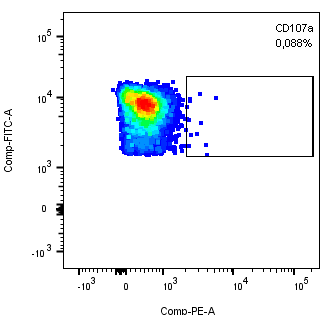

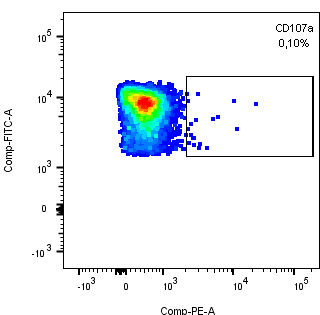

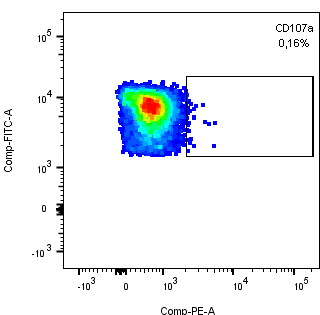

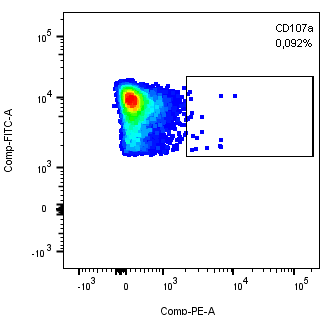

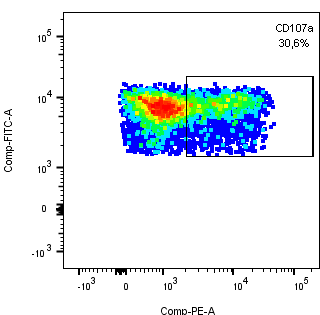


**B**

**RA patients**

**Healthy subjects**

**Supplementary Figure 7. CD8+ T-cell cytotoxic responses to HLA class I-presented peptides**. (A) Representative flow cytometry plots showing CD107a surface exposure in gated live, single CD3+CD8+ T cells from one rheumatoid arthritis (RA) patient and one healthy subject (HS) under unstimulated and peptide-stimulated conditions. (B) Graph showing the ratio of CD107a+ CD8+ T cells in stimulated over matched unstimulated cultures for each peptide. Boxes and whiskers indicate the median and range. Anti-CD3/anti-CD28 coated beads were used as positive control. Two viral peptide pools were used as stimulation controls: PHLA-A*02 and PHLA-B*08, with high affinity for HLA-A*02:01 and HLA-B*08:01 molecules, respectively. Group comparisons were performed using Mann-Whitney U test. *p < 0.05; **p < 0.01.
