# Supplementary material for: HLA class I-naturally presented synovial tissue peptides are recognized by CD8+ T lymphocytes from rheumatoid arthritis patients
Source: Front Immunol. 2026 Jul 3;17:1843318. doi: 10.3389/fimmu.2026.1843318 (PMC13375624; doi:10.3389/fimmu.2026.1843318)
Supplement: Supplementary file 2 [file SupplementaryFile2.docx]

**Supplementary Tables**

**Supplementary Table 1.** Main characteristics of recruited rheumatoid arthritis (RA) patients and healthy subjects (HS).

|  | **RA patients** | **HS** |
| --- | --- | --- |
| **Synovial fluid donors, n** | 15 | - |
| **Synovial tissue donors, n** | 2 | - |
| **Peripheral blood donors, n** | 25 | 25 |
| **Age, mean ± SD (years)** | 53.1 ± 11.2 | 50.3 ± 9.8 |
| **Gender, % of females / males** | 92 /8 | 76 / 24 |
| **RF, % of positive / negative / ND** | 92.0 / 8.0 / 0 | - |
| **Anti-CCP, % of positive / negative / ND** | 76.0 / 16.0 / 8.0 | - |
| **DAS28, mean ± SD** | 4.1 ± 1.1 | - |
| **Duration of disease, mean ± SD (years)** | 14.0 ± 11.0 | - |

Anti-CCP: anti-cyclic citrullinated peptide antibodies; DAS28: Disease Activity Score in 28 joints; ND: not determined; RF: rheumatoid factor; SD: standard deviation

**Supplementary Table 2.** Haplotypes and therapeutic scheme of rheumatoid arthritis (RA) patients and healthy subjects (HS) included in T-cell stimulation assays.

APAP: Acetaminophen; B: Betamethasone acetate; C: Celecoxib; DLX: Duloxetine; E: Etoricoxib; FOA: Folic acid; GOL: Golimumab; HCQ: Hydroxychloroquine; LEF: Leflunomide; MTX: Methotrexate; NA: not applicable; PRED: Prednisone; RTX: Rituximab; SSZ: Sulfasalazine; TOF: Tofacitinib; TOC: Tocilizumab; TRA: Tramadol; VitD: Vitamin D.

*Only those *HLA-ABC* allele groups expressed by either synovial tissue or monocyte donors were screened.

| Donor | *HLA-ABC* allele groups* | Treatment |
| --- | --- | --- |
| RA1805 | *A*01 A*03 B*08 C*04* | PRED / C / SSZ / APAP / HCQ |
| RA1912 | *A*02 A*24 B*39.1 B*51 C*01 C*07* | TOF |
| RA1925 | *A*02 C*05* | MTX / PRED / HCQ / C / TRA / APAP / FOA / Calcium-VitD |
| RA1956 | *A*02 B*39.1 C*05 C*06* | B / MTX / FOA / Calcium-VitD |
| RA2208 | *A*02 C*05* | MTX / PRED / C / LEF |
| RA2209 | *A*02 B*35 C*04 C*05* | MTX / HCQ / B |
| RA2301 | *A*26 B*08* | MTX / PRED / E / FOA |
| RA2302 | *A*01 A*02 B*08 B*51 C*01* | PRED / SSZ / APAP |
| RA2304 | *A*02 B*51 C*01 C*04* | FOA / C / DLX |
| RA2306 | *A*02 B*14 C*06 C*08* | GOL / MTX / LEF |
| RA2307 | *A*02 C*06 C*07* | PRED / MTX / RTX |
| RA2308 | *A*01 A*02 B*39.1 C*07* | RTX |
| RA2309 | *A*02 B*35 C*01 C*04* | TOC / PRED / HCQ |
| RA2310 | *A*02 A*24 B*39.1 C*04 C*07* | MTX / PRED |
| RA2311 | *A*02 B*35 C*04 C*05* | HCQ / MTX / PRED |
| RA2313 | *A*26 B*38 B*51* | MTX / LEF / HCQ / SSZ |
| RA2314 | *A*02 A*33 B*14 C*04 C*08* | HCQ / MTX / PRED /LEF / Calcium-VitD / FOA |
| RA2315 | *A*01 A*02 B*08 C*01 C*07* | LEF / MTX / PRED |
| RA2316 | *A*24 B*48 C*04 C*08* | HCQ / MTX / PRED / LEF / Calcium-VitD / FOA |
| RA2317 | *A*02 B*08 C*01 C*07* | MTX / FOA / Calcium-VitD |
| RA2318 | *A*01 B*08 B*51 C*07* | MTX / PRED |
| RA2319 | *A*02 B*51* | HCQ / PRED / C |
| RA2320 | *A*24 B*14 C*04 C*08* | MTX / LEF / PRED / GOL |
| RA2321 | *A*02 B*39.1 C*07* | LEF / HCQ / PRED |
| RA2322 | *A*02 A*26 B*38 B*39.1 C*07 C*12* | HCQ / PRED / LEF |
| HC006.22 | *A*26 B*08 B*38 C*07* | NA |
| HC023.23 | *A*01 A*24 B*08 C*07* | NA |
| HC028.23 | *A*02 B*35 C*04* | NA |
| HC029.23 | *A*02 B*51* | NA |
| HC030.23 | *A*02 C*07* | NA |
| HC030.24 | *A*01 A*02 B*58 C*12* | NA |
| HC031.23 | *A*01 B*08 B*39.1 C*07* | NA |
| HC3.24 | *A*02 B*44* | NA |
| HC5.24 | *A*02 B*51* | NA |
| HC6.24 | *A*01 A*02 B*08 C*07* | NA |
| HC7.24 | *A*03 B*51 C*05* | NA |
| HC8.24 | *A*01 A*02* | NA |
| HC9 | *A*02* | NA |
| HC9.24 | *A*03 B*39.1 C*07* | NA |
| HC10.24 | *B*39.1 C*07* | NA |
| HC13.24 | *A*24* | NA |
| HC15.24 | *A*02* | NA |
| HC1812 | *C*24* | NA |
| HC2324 | *A*02 B*14 B*51 C*08* | NA |
| HC32.25 | *B*39.1 C*04 C*07* | NA |
| HC3725 | *A*26 B*38 C*04* | NA |
| HC3825 | *B*35 B*38 C*04 C*12* | NA |
| HC3925 | *A*01 B*08 C*04 C*07* | NA |
| CS4125 | *A*02 A*24* | NA |
| CS4325 | *A*01 C*07* | NA |

**Supplementary Table 3.** *HLA-ABC* alleles and allele groups detected by polymerase chain reaction (PCR) using specific primers.

| ***Loci*** | **Sense primer** | **Anti-sense primer** | **Allele groups** | **Alleles included in each group** |
| --- | --- | --- | --- | --- |
|  | 5-TgCCAAgTggAgCACCCAA | 5-gCATCTTgCTCTgTgCAgAT |  | Internal control: third intron of the *HLA-DRB1* gene |
| **A** | 5-CgACgCCgCgAgCCAgAA | 5-AgCCCgTCCACgCACCg | *A*01* | **01:01; *01:02; *01:04N.* |
|  | 5-gTggATAgAgCAggAgggT | 5-CCAAgAgCgCAggTCCTCT | *A*02* | **02:01; *02:02; *02:03; *02:04; *02:05; *02:06; *02:07; *02:08; *02:09; *02:10; *02:11; *02:12; *02:13; *02:14; *02:15; *02:16; *02:17; *02:18; *02:19; *02:20; *02:21; *02:22; *02:23; *02:24; *02:25; *02:26.* |
|  | 5-AgCgACgCCgCgAgCCA | 5-CACTCCACgCACgTgCCA | *A*03* | **03:01; *03:02; *03:03N; *03:04.* |
|  | 5-ggCCggAgTATTgggACgA | 5-CCTCCAggTAggCTCTCTg | *A*24* | **24:02; *24:02102L; *24:03; *24:04; *24:05; *24:06; *24:07; *24:08; *24:09N; *24:10; *24:11N; *24:13; *24:14.* |
|  | 5-TCACAgACTgACCgAgCgAA | 5-ATgTAATCCTTgCCgTCgTAA | *A*26* | **26:01; *26:02; *26:04; *26:07; *26:08; *26:09; *43:01.* |
|  | 5-CCACTCCATgAggTATTTCAC | 5-gCCTTCACATTCCgTgTgTT | *A*33* | **33:01; *33:03.* |
|  | 5-gggTACCAgCAggACgCT | 5-gAgCCACTCCACgCACgT | *A*34* | **34:01; *34:02; *26:09.* |
| **B** | 5-gACCggAACACACAgATCTT | 5-CCgCgCgCTCCAgCgTg | *B*08* | **08:01; *08:02; *08:03.* |
|  | 5-AgCAggAggggCCggAA | 5-ggTCgCAgCCATACATCCA | *B*14* | **14:01; *14:02; *14:03; *14:04.* |
|  | 5-gACCggAACACACAgATCTT | 5-ggAggAAgCggCCgTCg | *B*35* | **35:01; *35:02; *35:03; *35:04; *35:05; *35:06; *35:07; *35:08; *35:091; *35:092; *35:11; *35:15; *35:17; *35:18; *35:19; *35:21; *53:01; *53:02.* |
|  | 5-ACCgAgAgAACCTgCggAT | 5-CgTgCCCTCCAggTAggT | *B*38* | **38:01 *38:021 *38:022.* |
|  | 5-CCgAgAgAgCCTgCggAA | 5-CgTgCCCTCCAggTAggT | *B*39.1* | **39:011; *39:013; *39:021; *39:022; *39:03; *39:04; *39:05; *39:061; *39:062; *39:07; *39:08; *39:09; *39:10; *39:11; *39:12; *67:011; *67:012.* |
|  | 5-gACCggAACACACAgATCTA | 5-CgTgCCCTCCAggTAggT | *B*39.2* | **39:10; *67:011; *67:012.* |
|  | 5-ggggAgCCCCgCTTCATT | 5-CgTCgTAgGCgTACTggTC | *B*44* | **35:19; *40:03; *40:09; *40:18; *44:02; *44:031; *44:032; *44:04; *44:05; *44:06; *44:07; *44:09.* |
|  | 5-CgCCgCgAgTCCgAgAgA | 5-CTCCAACTTgCgCTgggA | *B*48* | **40:12 *48:01 *48:03 *81:01.* |
|  | 5-ACCgAgAgAACCTgCggAT | 5-ATCCTTgCCgTCgTAgGCT | *B*49* | **49:01; 59:01.* |
|  | 5-ACCgAgAgAACCTgCggAT | 5-CgTTCAgggCgATgTAATCT | *B*51* | **51:011; *51:012; *51:021; *51:022; *51:03; *51:04; *51:05; *51:06; *51:07; *51:08; *51:09; *51:11N; *52:011; *52:012.* |
|  | 5-ACCgggAgACACAgATCTC | 5-CgTTCAgggCgATgTAATCT | *B*52* | **52:011; *52:012.* |
|  | 5-AACATgAAggCCTCCgCg | 5-gAggAggCggCCgTCg | *B*58* | **58:01; *58:02.* |
| **C** | 5-CACAgACTgACCgAgTgAg | 5-CCCCAggTCgCAgCCAC | *C*01* | **01:02; *01:03.* |
|  | 5-CCgAgTgAACCTgCggAAA | 5-gCCCCAggTCgCAgCCAA | *C*04* | **04:011; *04:012; *04:02; *04:03 *04:04; *18:01; *18:02.* |
|  | 5-CCgAgTgAACCTgCggAAA | 5-CgCgCgCTgCAgCgTCTT | *C*05* | **05:01.* |
|  | 5-TACTACAACCAgAgCgAggA | 5-ggTCgCAgCCATACATCCA | *C*06* | **06:02.* |
|  | 5-CCgCgggTATgACCAgTC | 5-CAgCCCCTCgTgCTgCAT | *C*07* | **07:01; *07:02; *07:03; *07:04; *07:05; *07:06; *07:07; *07:08.* |
|  | 5-ACgACACgCAgTTCgTgCA | 5-gCgCAggTTCCgCAggC | *C*08* | *Multiplex: 160bp only= *08:02. 160bp + 625bp= *08:01. *08:03.* |
|  | 5-CACAgACTgACCgAgTgA | 5-CCgCCgTgTCCgCggCA | *C*12* | **12:03.* |

**Supplementary Table 4.** List of antibodies and used in the different experiments described in the methodology.

| Antibody | Source | Catalogue Number | Clone | Supplier |
| --- | --- | --- | --- | --- |
| Purified anti-human HLA-ABC | Mouse IgG2a, k | BE0079 | W6/32 | Bio X Cell |
| PE anti-human CD86 | Mouse IgG2b, k | 305438 | IT2.2 | BioLegend |
| PECy7 anti-human CD40 | Mouse IgG1, k | 334322 | 5C3 | BioLegend |
| Alexa Fluor 647 anti-human CD11c | Mouse IgG1, k | 563787 | B-ly6 | BD Biosciences |
| Alexa Fluor 700 anti-human HLA-DR | Mouse IgG2a, k | 307610 | L243 | BioLegend |
| FITC anti-human CD1a | Mouse IgG1, κ | 300103 | HI149 | BioLegend |
| FITC anti-human CD14 | Mouse IgG1, κ | 325603 | HCD14 | BioLegend |
| BV605 anti-human CD80 | Mouse IgG1, k | 305225 | 2D10 | BioLegend |
| Pacific Blue anti-human CD1c | Mouse IgG1, k | 331508 | L161 | BioLegend |
| FITC anti-human CD8a | Mouse IgG1, k | 301006 | RPA-T8 | BioLegend |
| PE anti-human CD107a | Mouse IgG1, k | 328608 | H4A3 | BioLegend |
| PE-Cy7 anti-human IFN-y | Mouse IgG, k | 502528 | 4SB3 | BioLegend |
| APC anti-human CD3 | Mouse IgG, k | 344812 | SK7 | BioLegend |
| APC anti-human HLA-ABC | Mouse IgG2a, k | 11-9983-42 | W6/32 | Thermo Fisher Scientific |
| APC isotype control | Mouse IgG2a, k | 17-4724-81 | eBM2a | Thermo Fisher Scientific |
| Purified anti-human CD28 | Mouse IgG1, κ | 302902 | CD28.2 | BioLegend |
| Dynabeads™ anti-human CD3/CD28 for T cell expansion and activation | Mouse | 11161D | - | Thermo Fisher Scientific |

**Supplementary Table 5.** Peptides derived from viral proteins used as T-cell stimulation controls and their theoretical affinity for HLA-A*02:01 and HLA-B*08:02 molecules.

| Origin and protein name (amino acids in the protein sequence) | Sequence | Theoretical affinity for HLA class I molecules | |
| --- | --- | --- | --- |
|  |  | **HLA-A*02:01** | **HLA-B*08:01** |
| Epstein-Barr Virus, EBV-BMLF1 (259-267) | GLCTLVAML | SB | NB |
| Influenza A Virus, IFV-Matrix (58-66) | GILGFVFTL | SB | NB |
| Cytomegalovirus, CMV-pp65 (495-503) | NLVPMVATV | SB | NB |
| Influenza A Virus, IFV-NP (380-388) | ELRSRYWAI | NB | SB |
| Epstein-Barr Virus, EBV-EBNA3A (325-333) | FLRGRAYGL | WB | SB |

Peptides were assigned as strong binder (SB), weak binder (WB) or non-binder (NB) to the respective HLA class I molecule according to the NetMHCpan v4.2 classification.

**Supplementary Table 6.** HLA-ABC-associated peptides derived from non-housekeeping, inflammation-regulated proteins or with plausible relevance to rheumatoid arthritis.

| **Parental protein** | **Peptide name** | **Sequence** | **Source sample(s)** | **Donor HLA class I molecule(s) to which the peptide is predicted as strong binder.** | **Study-relevant HLA class I molecule(s) to which the peptide is predicted as strong binder.** |
| --- | --- | --- | --- | --- | --- |
| Acidic leucine-rich nuclear phosphoprotein 32 family member A | ANP32A | **FLSTINVGL** | ST2 | A*02:01 | A*02:01 |
| Alpha-crystallin B chain | CRYAB | WFDTGLSEM | ST1 | C*04:01 | C*07 |
| Annexin A2 | ANXA2 | ALSGHLETV | ST2 | NA | A*02:01 |
| Annexin A5 | ANXA5 | DAYELKHAL | ST1 and ST2 | B*35:01; B*39:09; B*51:01 | B*08:01; C*07 |
| Aryl hydrocarbon receptor | AHR | **ILPPQLALF** | ST2 | A*24:02; C*01:02 | C*07 |
| Bromodomain-containing protein 4 | BRD4 | FAADVRLMF | ST1 | B*35:01; C*04:01; C*07:01 | C*07 |
| Cathepsin D | CTSD | IPLHKFTSI | ST2 | B*51:01 | B*08:01 |
|  |  | **YLSQDTVSV** | ST2 | A*02:01 | A*02:01 |
|  |  | YPRISVNNV | ST2 | B*51:01 | B*08:01 |
| Complement C3 | C3 | AYVVKVFSL | ST2 | A*24:02; C*07:02 | B*08:01; C*07 |
| Dipeptidyl peptidase 2 | DPP7 | AYDTVRWEF | ST2 | A*24:02; C*07:02 | C*07 |
| Exportin-1 | XPO1 | **VLIDYQRNV** | ST2 and SF-DC3 | A*02:01 | A*02:01 |
|  |  | VYIGKLNMI | ST2 | A*24:02; C*07:02 | C*07 |
| Fibrinogen alpha chain | FGA | FFSPMLGEF | ST1 and ST2 | C*04:01; C*07:01; A*24:02; C*07:02 | C*07 |
|  |  | TASDFITKM | ST1 | B*35:01; C*07:01 | C*07 |
| Gelsolin | GSN | YPKQTQVSV | ST2 | B*51:01 | B*08:01 |
| Heat shock factor protein 1 | HSF1 | ALWREVASL | ST2 | A*02:01 | A*02:01 |
| Heterogeneous nuclear ribonucleoproteins A2/B1 | HNRNPA2B1 | FGPGPGSNF | ST2 | C*01:02 | C*07 |
| Histone deacetylase 1 | HDAC1 | DYIKFLRSI | ST2 | A*24:02 | C*07 |
|  |  | PYNDYFEYF | ST2 | A*24:02; C*07:02 | C*07 |
| Hypoxia-inducible factor 1-alpha | HIF1A | FYLKALDGF | ST2 | A*24:02; C*07:02 | C*07 |
|  |  | IQHDLIFSL | ST2 | A*02:01; B*39:09; C*07:02 | A*02:01; C*07 |
| Lamin-A | LMNA | VYIDKVRSL | ST2 | A*24:02; C*01:02; C*07:02 | B*08:01; C*07 |
| Macrophage migration inhibitory factor | MIF | **FLSELTQQL** | ST2 | A*02:01; B*39:09; C*01:02; C*07:02 | A*02:01; B*08:01; C*07 |
| Nuclear autoantigen Sp-100 | SP100 | LYDIVFKHF | ST2 | A*24:02; C*07:02 | C*07 |
| Nuclear receptor coactivator 3 | NCOA3 | ALLDQLHTL | ST2 | A*02:01; C*01:02 | A*02:01; C*07 |
| Progressive ankylosis protein homolog | ANKH | **SISDVIAQV** | ST2 | A*02:01 | A*02:01; C*07 |
| Protein C-ets-1 | ETS1 | **ILWEHLEIL** | ST2 | A*02:01 | A*02:01 |
| Raftlin-2 | RFTN2 | **SILDIVTKV** | ST2 | A*02:01 | A*02:01; C*07 |
| Serine/threonine-protein kinase/endoribonuclease IRE1 | IRE1α | TLDGSLHAV | ST2 | A*02:01 | A*02:01; C*07 |
| Upstream stimulatory factor 1 | USF1 | **FPDPNVKYV** | ST1 | B*35:01; C*04:01 | C*07 |
| Vimentin | VIM | SLNLRETNL | ST2 | NA | B*08:01 |
|  |  | **SLQEEIAFL** | ST2 | A*02:01; C*01:02 | A*02:01 |
|  |  | WYKSKFADL | ST2 | NA | B*08:01 |

For each peptide, the table indicates the parental protein, source sample(s), and the donor-derived or study-relevant HLA class I molecule(s) to which they were predicted as strong binders (SB) according to the NetMHCpan v4.2 classification. NA indicates that the peptide was not assigned as SB for any donor-derived or study-relevant HLA molecule. The study-relevant HLA class I molecules considered were HLA-A*02:01, HLA-B*08:01, and HLA-A*C07. Grey shading denotes the ten proteins selected after a higher-stringency mechanistic prioritization. When more than one peptide from the same parental protein met the selection criteria, the peptide highlighted in grey and shown in bold was selected for subsequent functional assays. The peptide name corresponds to the parental protein gene symbol or commonly used protein abbreviation according to UniProt.

**Supplementary Table 7.** Functional, subcellular, expression-pattern, and RA-related annotation of the ten prioritized parental proteins selected for CD8+ T-cell stimulation assays.

| **Parental protein** | **Peptide name** | **Biological process** | **Subcellular localization** | **Expression pattern** | **Association with RA / functional relevance** |
| --- | --- | --- | --- | --- | --- |
| Acidic leucine-rich nuclear phosphoprotein 32 family member A | ANP32A | Signaling | Nucleus; cytoplasm; cytosol; intracellular structures; endosome/ lysosome; endoplasmic reticulum | Secreted in plasma; tissue/cell enriched annotation: thymus; ubiquitous; widely expressed | Reported target of autoantibodies |
| Aryl hydrocarbon receptor | AHR | Biological regulation; response to stimulus | Nucleus; cytoplasm; cytosol; endoplasmic reticulum | Secreted in plasma; ubiquitous; widely expressed | Reported in RA-related tissues/cells; functionally implicated in RA inflammation |
| Cathepsin D | CTSD | Metabolic process | Endoplasmic reticulum; Golgi apparatus | Secreted in plasma; ubiquitous | Increased/activated in RA-related tissues/cells |
| Exportin-1 | XPO1 | Metabolic process; cellular component organization or biogenesis | Nucleus; cytosol; endosome/lysosome | Secreted in plasma; ubiquitous; widely expressed | Reported in RA-related tissues/cells; functionally implicated in RA-related inflammatory pathways |
| Macrophage migration inhibitory factor | MIF | Biological regulation; localization; signaling; immune system process | Nucleus; cytosol; intracellular structures; endoplasmic reticulum; Golgi apparatus; extracellular region; cell membrane | Secreted in plasma; ubiquitous; widely expressed | Reported in RA-related tissues/cells; functionally implicated in RA inflammation |
| Progressive ankylosis protein homolog | ANKH | Metabolic process; immune system process | Nucleus; cytosol; intracellular structures; extracellular region | Tissue/cell enriched (spleen, Langerhans cells); secreted in plasma; ubiquitous; widely expressed | Reported in RA-related tissues/cells; functionally implicated in arthritis-related tissue damage |
| Protein C-ets-1 | ETS1 | Biological regulation; other | Nucleus; cytosol; cell membrane | Tissue/cell enriched (monocytes); secreted in plasma; widely expressed | Increased in RA-related tissues/cells; functionally implicated in RA inflammation and arthritis-related tissue damage |
| Raftlin-2 | RFTN2 | Biological regulation; cellular component organization or biogenesis; signaling | Cytosol; intracellular structures; endosome/lysosome; cell membrane | Tissue/cell enriched (memory B cells) | Reported in RA-related tissues/cells; functionally implicated in immune pathways relevant to RA |
| Upstream stimulatory factor 1 | USF1 | Metabolic process; signaling | Nucleus; intracellular structures; extracellular region; cell membrane; cytoskeleton | Tissue/cell enriched (non-classical monocytes); ubiquitous; widely expressed | Reported in RA-related tissues/cells; functionally implicated in immune/inflammatory regulation |
| Vimentin | VIM | Cellular component organization or biogenesis | Nucleus | Widely expressed; ubiquitous | Target of autoantibodies; target of T-cell responses |

**Supplementary Table 8.** CD8+ T-cell responsiveness of rheumatoid arthritis patients to selected peptides and the theoretical binding assignment of those peptides to HLA-ABC molecules carried by each patient.

| Patients / HLA-ABC molecules | Response to peptides and theoretical binding assignment to HLA-ABC molecules | | | | | | | | | | |
| --- | --- | --- | --- | --- | --- | --- | --- | --- | --- | --- | --- |
|  | **ANP32A** | **XPO1** | **MIF** | **RFTN2** | **CATD** | **ANKH** | **ETS1** | **USF1** | **VIM** | **AHR** |  |
| RA1805  A*01 A*03 B*08 C*04 | C*04 WB  (-) | C*04 WB  (-) | B*08 SB C*04 SB  (-) | C*04 WB  (+) | B*08 WB C*04 SB  (+) | C*04 WB  (+) | B*08 WB C*04 WB  (-) | A*01 WB B*08 SB C*04 SB  (-) | C*04 SB  (-) | C*04 SB  (-) |  |
| RA1912  A*02 A*24 B*39.1 B*51 C*01 C*07 | A*02 SB B*39.1 WB C*01 WB  (-) | A*02 SB B*39.1 WB C*01 WB C*07 WB  (+++) | A*24 WB  A*02 SB  B*39.1 SB  C*01 SB  C*07 SB  (+++) | A*02 SB B*51 WB C*01 WB C*07 WB  (-) | A*02 SB B*39.1 WB C*01 WB C*07 WB  (-) | A*02 SB B*51 WB C*01 WB C*07 WB  (-) | A*02 SB C*01 WB C*07 WB  (+) | A*02 WB B*39.1 SB  B*51 SB C*01 WB C*07 WB  (+++) | A*02 SB C*01 SB C*07 WB  (+) | A*02 WB  A*24 SB  C*01 SB  C*07 SB  (-) |  |
| RA1925  A*02 C*05 | A*02 SB C*05 WB  (-) | A*02 SB C*05 WB  (+) | A*02 SB  C*05 SB  (-) | A*02 SB C*05 WB  (-) | A*02 SB C*05 SB  (-) | A*02 SB C*05 SB  (-) | A*02 SB C*05 WB  (-) | A*02 WB C*05 SB  (+++) | A*02 SB C*05 SB  (-) | A*02 WB C*05 WB  (+++) |  |
| RA1956  A*02 B*39.1 C*05 C*06 | A*02 SB B*39.1 WB C*05 WB  (-) | A*02 SB C*05 WB  C*06 SB  (-) | A*02 SB  B*39.1 SB  C*05 SB  C*06 SB  (++) | A*02 SB B*39.1 WB C*05 WB  C*06 WB  (-) | A*02 SB B*39.1 WB C*05 SB  C*06 WB  (-) | A*02 SB C*05 SB  C*06 WB  (+) | A*02 SB C*05 WB  (-) | A*02 WB B*39.1 SB C*05 SB  C*06 WB  (++) | A*02 SB C*05 SB  (++) | A*02 WB C*05 WB  C*06 WB  (-) |  |
| RA2208  A*02 C*05 | A*02 SB C*05 WB  (+++) | A*02 SB C*05 WB  (+++) | A*02 SB  C*05 SB  (+++) | A*02 SB C*05 WB  (+++) | A*02 SB C*05 SB  (+++) | A*02 SB C*05 SB  (++) | A*02 SB C*05 WB  (+++) | A*02 WB C*05 SB  (+++) | A*02 SB C*05 SB  (+++) | A*02 WB C*05 WB  (+++) |  |
| RA2209  A*02 B*35 C*04 C*05 | A*02 SB C*04 WB C*05 WB  (-) | A*02 SB C*04 WB C*05 WB  (+) | A*02 SB B*35 WB C*04 SB C*05 SB  (-) | A*02 SB C*04 WB C*05 WB  (-) | A*02 SB C*04 SB C*05 SB  (-) | A*02 SB C*04 WB C*05 SB  (-) | A*02 SB C*04 WB C*05 WB  (-) | A*02 WB B*35 SB C*04 SB C*05 SB  (+++) | A*02 SB C*04 SB C*05 SB  (-) | A*02 WB C*04 SB C*05 WB  (++) |  |
| RA2301  A*26 B*08 | NB  (++) | NB  (-) | A*26 WB  B*08 SB  (+) | A*26 WB  (+) | B*08 WB  (-) | A*26 SB  (+) | B*08 WB  (+) | B*08 SB  (++) | NB  (++) | A*26 WB  (++) |  |
| RA2302  A*01 A*02 B*08 B*51 C*01 | A*02 SB C*01 WB  (-) | A*02 SB C*01 WB  (+) | A*02 SB  B*08 SB  C*01 SB    (+++) | A*02 SB B*51 WB C*01 WB  (+) | A*02 SB B*08 WB C*01 WB  (-) | A*02 SB B*51 WB C*01 WB  (-) | A*02 SB B*08 WB C*01 WB  (-) | A*01 WB A*02 WB B*08 SB B*51 WB C*01 WB  (+++) | C*01 SB  A*02 SB  (+) | A*02 WB C*01 SB  (+) |  |
| RA2304  A*02 B*51 C*01 C*04 | A*02 SB C*01 WB  C*04 WB  (-) | A*02 SB C*01 WB  C*04 WB  (-) | A*02 SB  C*01 SB  C*04 SB  (-) | A*02 SB  B*51 WB C*01 WB  C*04 WB  (-) | A*02 SB C*01 WB  C*04 SB  (-) | A*02 SB  B*51 WB C*01 WB  C*04 WB  (-) | A*02 SB C*01 WB  C*04 WB  (+++) | A*02 WB  B*51 SB  C*01 WB  C*04 SB  (-) | A*02 SB C*01 SB  C*04 SB  (-) | A*02 WB C*01 SB  C*04 SB  (+) |  |
| RA2306  A*02 B*14 C*06 C*08 | A*02 SB C*08 WB  (-) | A*02 SB C*06 SB  C*08 WB  (++) | A*02 SB  B*14 WB  C*06 SB  C*08 SB  (+++) | A*02 SB C*06 WB  C*08 WB  (+) | A*02 SB C*06 WB  C*08 SB  (+++) | A*02 SB C*06 WB  C*08 SB  (+) | A*02 SB C*08 WB  (-) | A*02 WB  B*14 WB C*06 WB  C*08 SB  (+) | A*02 SB  C*08 WB  (+) | A*02 WB  (+++) |  |
| RA2307  A*02 C*06 C*07 | A*02 SB C*07 WB  (-) | A*02 SB C*06 SB  C*07 WB  (-) | A*02 SB  C*06 SB  C*07 SB  (-) | A*02 SB C*06 WB  C*07 SB  (-) | A*02 SB C*06 WB  C*07 WB  (-) | A*02 SB C*06 WB  C*07 WB  (-) | A*02 SB  C*07 WB  (-) | A*02 WB C*06 WB  C*07 WB  (-) | A*02 SB  C*07 WB  (-) | A*02 WB  C*06 WB  C*07 SB  (-) |  |
| RA2308  A*01 A*02 B*39.1 C*07 | A*02 SB  B39.1 WB  C*07 WB  (-) | A*02 SB  C*07 WB  (+++) | A*02 SB  B39.1 SB  C*07 SB  (-) | A*02 SB  C*07 WB  (-) | A*02 SB  B39.1 WB  C*07 WB  (-) | A*02 SB  C*07 WB  (+) | A*02 SB  C*07 WB  (-) | A*01 WB  A*02 WB  B39.1 SB  C*07 WB  (+++) | A*02 SB  C*07 WB  (-) | A*02 WB  C*07 SB  (+++) |  |
| RA2309  A*02 B*35 C*01 C*04 | A*02 SB  C*01 WB C*04 WB  (+) | A*02 SB  C*01 WB C*04 WB  (-) | A*02 SB  B*35 WB  C*01 SB  C*04 SB  (++) | A*02 SB  C*01 WB C*04 WB  (+) | A*02 SB  C*01 WB C*04 SB  (++) | A*02 SB  C*01 WB C*04 WB  (+++) | A*02 SB  C*01 WB C*04 WB  (++) | A*02 WB  B*35 SB  C*01 WB C*04 SB  (+) | A*02 SB  C*01 SB C*04 SB  (+) | A*02 WB  C*01 SB C*04 SB  (++) |  |
| RA2310  A*02 A*24 B*39.1 C*04 C*07 | A*02 SB B*39.1 WB  C*04 WB  (-) | A*02 SB  C*04 WB  C*07 WB  (-) | A*02 SB  A*24 WB  C*04 SB  C*07 SB  (-) | A*02 SB  C*04 WB  C*07 WB  (-) | A*02 SB  B*39.1 WB  C*04 SB  (-) | A*02 SB  C*04 WB  C*07 WB  (-) | A*02 SB  C*04 WB  (-) | A*02 WB  B*39.1 SB  C*04 SB  C*07 SB  (+) | A*02 SB  C*04 SB  (-) | A*02 WB  A*24 SB  C*04 SB  C*07 WB  (-) |  |
| RA2311  A*02 B*35 C*04 C*05 | A*02 SB  C*04 WB  C*05 WB  (+++) | A*02 SB  C*04 WB  C*05 WB  (+) | A*02 SB  B*35 WB  C*04 SB  C*05 SB  (-) | A*02 SB  C*04 WB  C*05 WB  (-) | A*02 SB  C*04 SB  C*05 SB  (-) | A*02 SB  C*04 WB  C*05 SB  (+) | A*02 SB  C*04 WB  C*05 WB  (+) | A*02 WB  B*35 SB  C*04 SB  C*05 SB  (+++) | A*02 SB  C*04 SB  C*05 SB  (-) | A*02 WB  C*04 SB  C*05 WB  (+++) |  |
| RA2313  A*26 B*38 B*51 | NB  (-) | NB  (-) | A*26 WB  B*38 WB  (+) | A*26 WB  B*51 WB  (+++) | NB  (+++) | A*26 SB  B*51 WB  (+) | NB  (++) | B*38 WB  (-) | NB  (+) | A*26 WB  (+++) |  |
| RA2314  A*02 A*33 B*14 C*04 C*08 | A*02 SB  C*04 WB  C*08 WB  (-) | A*02 SB  C*04 WB  C*08 WB  (-) | A*02 SB  B*14 WB  C*04 SB  C*08 SB  (-) | A*02 SB  C*04 WB  C*08 WB  (-) | A*02 SB  C*04 SB  C*08 SB  (-) | A*02 SB  C*04 WB  C*08 SB  (-) | A*02 SB  C*04 WB  C*08 WB  (+) | A*02 WB  B*14 WB  C*04 SB  C*08 SB  (-) | A*02 SB  C*04 SB  C*08 WB  (-) | A*02 WB  C*04 SB  C*08 WB  (++) |  |
| RA2315  A*01 A*02 B*08 C*01 C*07 | A*02 SB  C*01 WB  C*07 WB  (-) | A*02 SB  C*01 WB  C*07 WB  (-) | A*02 SB  B*08 SB  C*01 SB  C*07 SB  (-) | A*02 SB  C*01 WB  C*07 WB  (-) | A*02 SB  B*08 WB  C*01 WB  C*07 WB  (-) | A*02 SB  C*01 WB  C*07 WB  (-) | A*02 SB  B*08 WB  C*01 WB  C*07 WB  (+) | A*01 WB  A*02 WB  B*08 SB  C*01 WB  C*07 SB  (-) | A*02 SB  C*01 SB  C*07 WB  (-) | A*02 WB  C*01 SB  C*07 SB  (-) |  |
| RA2316  A*24 B*48 C*04 C*08 | B*48 WB  C*04 WB  C*08 WB  (-) | C*04 WB  C*08 WB  (-) | A*24 WB  B*48 SB  C*04 SB  C*08 SB  (-) | B*48 WB  C*04 WB  C*08 WB  (-) | B*48 WB  C*04 SB  C*08 SB  (-) | C*04 WB  C*08 SB  (-) | B*48 SB  C*04 WB  C*08 WB  (-) | C*04 SB  C*08 SB  (-) | B*48 WB  C*04 SB  C*08 WB  (-) | A*24 SB  C*04 SB  C*08 WB  (-) |  |
| RA2317  A*02 B*08 C*01 C*07 | A*02 SB  C*01 WB  C*07 WB  (+++) | A*02 SB  C*01 WB  C*07 WB  (+++) | A*02 SB  B*08 SB  C*01 SB  C*07 SB  (+++) | A*02 SB  C*01 WB  C*07 WB  (+) | A*02 SB  B¨08 WB  C*01 WB  C*07 WB  (-) | A*02 SB  C*01 WB  C*07 WB  (+++) | A*02 SB  B*08 WB  C*01 WB  C*07 WB  (+++) | A*02 WB  B*08 SB  C*01 WB  C*07 WB  (+++) | A*02 SB  C*01 SB  C*07 WB  (+) | A*02 WB  C*01 SB  C*07 SB  (-) |  |
| RA2318  A*01 B*08 B*51 C*07 | C*07 WB  (-) | C*07 WB  (-) | B*08 SB  C*07 SB  (+) | B*51 WB  C*07 SB  (-) | B*08 WB  (+) | B*51 WB  C*07 WB  (-) | B*08 WB  C*07 SB  (-) | A*01 WB  B*08 SB  B*51 SB  C*07 SB  (-) | C*07 WB  (-) | C*07 SB  (-) |  |
| RA2319  A*02 B*51 | A*02 SB  (+++) | A*02 SB  (+++) | A*02 SB  (+++) | A*02 SB  B*51 WB  (+++) | A*02 SB  (+++) | A*02 SB  B*51 WB  (+++) | A*02 SB  (+++) | A*02 WB  B*51 SB  (+++) | A*02 SB  (+++) | A*02 WB  (+++) |  |
| RA2320  A*24 B*14 C*04 C*08 | C*04 WB  C*08 WB  (-) | C*04 WB  C*08 WB  (-) | A*24 WB B*14 WB C*04 SB  C*08 SB  (+) | C*04 WB  C*08 WB  (-) | C*04 SB  C*08 SB  (-) | C*04 WB  C*08 SB  (-) | C*04 WB  C*08 WB  (-) | B*14 WB C*04 SB  C*08 WB  (-) | C*04 SB  C*08 WB  (+) | A*24 SB  C*04 SB  C*08 WB  (+) |  |
| RA2321  A*02 B*39.1 C*07 | A*02 SB  B*39.1 WB  C*07 WB  (+++) | A*02 SB  C*07 WB  (+) | A*02 SB  B*39.1 SB  C*07 SB  (-) | A*02 SB  C*07 WB  (-) | A*02 SB  B*39.1 WB  C*07 WB  (-) | A*02 SB  C*07 WB  (-) | A*02 SB  C*07 WB  (+) | A*02 WB  B*39.1 SB  C*07 SB  (-) | A*02 SB  C*07 WB  (+) | A*02 WB  C*07 WB  (+) |  |
| RA2322  A*02 A*26 B*38 B*39.1 C*07 C*12 | A*02 SB  B*39.1 WB  C*07 WB  (++) | A*02 SB  C*07 WB  C*12 WB  (-) | A*02 SB  A*26 WB  B*38 WB  C*07 SB  C*12 SB  (-) | A*02 SB  A*26 WB  C*07 WB  C*12 SB  (-) | A*02 SB  C*07 WB  C*12 WB  (-) | A*02 SB  A*26 SB  C*07 WB  C*12 SB  (-) | A*02 SB  C*07 WB  C*12 WB  (-) | A*02 WB  B*38 WB  C*07 SB  C*12 WB  (+++) | A*02 SB  C*07 WB  (+) | A*02 WB  A*26 WB  C*07 WB  C*12 WB  (+++) |  |

Rheumatoid arthritis patients were classified as responders when their stimulated/unstimulated IFN-γ+CD107a+CD8+ T-cell ratio exceeded the 75th percentile (P75) of the healthy subjects group IFN-γ+CD107a+CD8+ T-cell ratio distribution for that peptide. (-): Ratio < P75; (+): Ratio ≥ P75 and ≤ 2 fold P75; (++): Ratio >2 fold and ≤ 3 fold P75; (+++): Ratio >3 fold P75. Each peptide was assigned as strong binder (SB), weak binder (WB) or non-binder (NB) to HLA class I molecules carried by each patient according to the NetMHCpan v4.2 classification. NB is only shown when the peptide was NB for all HLA-ABC molecules assessed. Gray shaded cells indicate responder patients carrying at least one HLA-ABC molecule for which the corresponding peptide was assigned as SB.

**Supplementary Table 9**: Frequency of individuals carrying at least one predicted strong-binding (SB) HLA-ABC molecule for each peptide in RA patients and healthy subjects (HS).

Each peptide was assigned as strong binder to HLA class I molecules carried by each individual according to the NetMHCpan v4.2 classification. Fisher’s exact test (*p<0.05).

| Peptide name | % RA patients carrying a SB HLA-ABC molecule (n) | % HS carrying a SB HLA-ABC molecule (n) | *p*-value |
| --- | --- | --- | --- |
| ANP32A | 76.0 (19) | 48.0 (12) | 0.0792 |
| XPO1 | 76.0 (19) | 48.0 (12) | 0.0792 |
| MIF | 96.0 (24) | 96.0 (24) | >0.9999 |
| RFTN2 | 80.0 (20) | 52.0 (13) | 0.0718 |
| CATD | 88.0 (22) | 72.0 (18) | 0.2890 |
| ANKH | 92.0 (23) | 64.0 (16) | *0.0374 |
| ETS1 | 84.0 (21) | 48.0 (12) | *0.0054 |
| USF1 | 92.0 (23) | 72.0 (18) | 0.1383 |
| VIM | 88.0 (22) | 76.0 (19) | 0.4635 |
| AHR | 64.0 (16) | 52.0 (13) | 0.5672 |
